# Autism‐related language preferences of English‐speaking individuals across the globe: A mixed methods investigation

**DOI:** 10.1002/aur.2864

**Published:** 2022-12-06

**Authors:** Connor Tom Keating, Lydia Hickman, Joan Leung, Ruth Monk, Alicia Montgomery, Hannah Heath, Sophie Sowden

**Affiliations:** ^1^ School of Psychology University of Birmingham Birmingham UK; ^2^ School of Psychology University of Auckland Auckland New Zealand; ^3^ Autistic member of the Autism New Zealand Community Advisory Group, New Zealand/School of Medical Sciences University of Auckland Auckland New Zealand; ^4^ School of Psychiatry University of New South Wales Sydney New South Wales Australia

**Keywords:** autism, disability, language, neurodiversity, policy, terminology

## Abstract

Over the past two decades, there have been increasing discussions around which terms should be used to talk about autism. Whilst these discussions have largely revolved around the suitability of identity‐first language and person‐first language, more recently this debate has broadened to encompass other autism‐related terminology (e.g., ‘high‐functioning’). To date, academic studies have not investigated the language preferences of autistic individuals outside of the United Kingdom or Australia, nor have they compared levels of endorsement across countries. Hence, the current study adopted a mixed‐methods approach, employing both quantitative and qualitative techniques, to explore the linguistic preferences of 654 English‐speaking autistic adults across the globe. Despite variation in levels of endorsement between countries, we found that the most popular terms were similar—the terms ‘Autism’, ‘Autistic person’, ‘Is autistic’, ‘Neurological/Brain Difference’, ‘Differences’, ‘Challenges’, ‘Difficulties’, ‘Neurotypical people’, and ‘Neurotypicals’ were consistently favored across countries. Despite relative consensus across groups, both our quantitative and qualitative data demonstrate that there is no universally accepted way to talk about autism. Our thematic analysis revealed the reasons underlying participants’ preferences, generating six core themes, and illuminated an important guiding principle—to respect personal preferences. These findings have significant implications for informing practice, research and language policy worldwide.

## Lay Summary

There has been a lot of debate about what language should be used to talk about autism. In our study, we assessed the language preferences of 654 English‐speaking autistic people from multiple different countries. Based on our results, we recommend that individuals ask the autistic people they interact with (e.g., the autistic people they are working with, autistic friends or family members, etc.) about their preferences and use those terms. If in doubt, individuals should use the most popular terms (e.g., ‘Autism’, ‘Autistic person’, ‘Is autistic’, ‘Neurological/Brain Difference’, ‘Differences’, ‘Challenges’, ‘Difficulties’, ‘Neurotypical people’, and ‘Neurotypicals’), and be mindful of the ideas underlying language choices (e.g., avoiding ableist language).

## INTRODUCTION

Over the past two decades, there have been increasing discussions around what terms should be used to talk about autism spectrum disorder (ASD; hereafter ‘autism’). Primarily, these discussions have involved autistic activists and scholars debating the suitability of person‐first (‘person with autism’) and identity‐first (i.e., ‘autistic person’) language. Person‐first language, which is often used in clinical and research environments (e.g., Crocker & Smith, 2019), is argued to place significance on the person rather than their disability by acknowledging a distinct separation (Maio, 2001; Wright, 1983). However, many individuals argue that, by emphasizing this separation, person‐first language inadvertently accentuates stigma (see Botha et al., 2021; Gernsbacher, 2017), perpetuating the notion that autism is a ‘defect’ that must be removed from the individual (and indirectly suggesting that disability is inherently bad; Andrews et al., 2019; Jernigan, 2009; La Forge, 1991; Vaughan, 2009; Botha et al., 2021). In contrast, identity‐first language, which is recommended by some autism bodies (e.g., Autistica, Sterry, 2019) and academic journals (e.g., Autism in Adulthood, 2019), is said to acknowledge autism as a core part of someone's identity, just like their gender or ethnicity (e.g., Sinclair, 2013). Since many individuals believe that an autistic person can never, and should never be separated from their autism, many activists endorse identity‐first language (e.g., ‘autistic person’; Brown, 2011; Halmari, 2011; Sinclair, 2013).

Whilst debate is rife regarding person‐first and identity‐first language amongst activists and scholars, until relatively recently no academic studies had explored the preferences of autistic individuals with respect to identifying language. The first study to do so found that larger numbers of autistic adults in the United Kingdom (UK) endorsed ‘autistic’ and ‘autistic person’ (i.e., identity‐first language) than ‘person with autism’ (i.e., person‐first language; Kenny et al., 2016). Interestingly these findings are echoed in Australian samples, with autistic participants rating the terms ‘autistic’, ‘person on the spectrum’ and ‘autistic person’ significantly higher than ‘person with autism’, ‘person with ASD’, and ‘person with Autism Spectrum Condition (ASC)’ (Bury et al., 2020). However, it is important to note that whilst these terms were most preferred by the autistic community, this stance was by no means universal^1^—approximately 40% of autistic individuals in the United Kingdom did not endorse ‘autistic’ and over 50% did not endorse ‘autistic person’ (Kenny et al., 2016). As Robison (2019) highlights, ‘language that is appropriate to one person is offensive to another’ (p. 1006).

Although discussions have largely centred around the adequacy of person‐first and identity‐first language, there are many other terms that are discussed within the autism community. For example, functioning‐level descriptors such as ‘high‐functioning’ and ‘low‐functioning’ are endorsed by very few autistic adults in the United Kingdom (‘high‐functioning’: ~20%; ‘low‐functioning’: <10%; Kenny et al., 2016). Despite opposition from autistic adults (Kenny et al., 2016; Ortega, 2009) and some autism researchers (who argue these terms are imprecise and ‘hinder scientific progress’; Bal et al., 2017), these descriptors are often used in academic publications (Bal et al., 2017). Indeed, a search on this Journal's website (*Autism Research*) yielded 158 results for ‘high‐functioning’ and 154 results for ‘low‐functioning’ in 2021 alone. Rather than using these descriptors, Bottema‐Beutel et al. (2021) suggest that one should describe the specific strengths and needs of autistic people, and acknowledge that the level of support needs likely varies across domains.

In addition to offering insight on functioning labels, this group of researchers provided comprehensive guidelines for avoiding ableist language across a broad range of terminology (Bottema‐Beutel et al., 2021). In terms of identifying language, Bottema‐Beutel et al. (2021) endorse the use of identity‐first language, or ‘on the autism spectrum’, echoing the thoughts of the autistic community. Furthermore, they highlight that we must move away from interpreting any differences between autistic and non‐autistic groups as evidence of autistic ‘deficits’, and instead consider that autistic people may have relative strengths over non‐autistic people, or that differences between groups have neutral value unless actively demonstrated otherwise. In addition, before this publication, no literature had addressed how we refer to non‐autistic people. However, consideration is important here as the terms we use to speak about non‐autistic people inherently have connotations about autism and autistic people. For example, if someone refers to a non‐autistic person as a ‘healthy control’, they are endorsing a medical model of autism, and indirectly suggesting that autistic people are unhealthy or sick. Instead, Bottema‐Beutel et al. (2021) encourage the use of ‘non‐autistic’, or ‘neurotypical’ if there has been extensive screening to rule out most forms of neurodivergence. Whilst a group of researchers have suggested that these are the most appropriate terms for referring to non‐autistic people, studies are yet to consider the preferences of autistic individuals themselves. In addition, studies have not yet explored the preferences of *autistic individuals* for conceptualizing autism more broadly (i.e., is autism a ‘condition’, ‘disability’, ‘disorder’, or ‘neurological difference’?), or for referring to the difficulties of autistic individuals (e.g., ‘deficits’, or ‘differences’, etc.). However, such investigations should be a priority given that autistic individuals are part of a marginalized and vulnerable group (Bury et al., 2020), and it is their voices that should lead the way.

In addition, the studies to date are informative with respect to the language preferences of those in the United Kingdom and Australia, however, we should be cautious about attributing these preferences to the global autistic population, especially as social and ideological beliefs influence language use (Kenny et al., 2016) and these beliefs differ between cultures. Cross‐cultural variation in language preferences could manifest in a number of ways. For example, there could be variation in preferences for terms that are *widely used* across the globe, such as ‘autistic person’ or ‘person with autism’, or it could be that certain cultures employ unique terms to conceptualize autism that are not often used elsewhere. For instance, there are multiple te reo Māori (the language of the indigenous population of New Zealand) terms for conceptualizing autism, such as Takiwātanga and Kura Urupare.^2^ Further work is needed to understand the language preferences of autistic people from diverse racial, ethnic, linguistic, and cultural backgrounds (Bottema‐Beutel et al., 2021). Such work should explore the preferences of a diverse set of autistic individuals and compare these using standardized questionnaires (with open text boxes for additional responses). By taking this approach, citizens, researchers and governments can ensure that they use the terminology that is most preferred within their region, thus reducing stigmatization and marginalization, and facilitating the formation of constructive alliances (Dunn & Andrews, 2015).

The current study adopted a mixed‐methods approach to investigate the preferences of autistic adults across the globe on a broad range of autism‐related terminology. To address this aim, autistic participants from multiple countries (e.g., Australia, Canada, Ireland, New Zealand, South Africa, UK, USA, etc.) completed a language preferences questionnaire including both quantitative and qualitative components. Here, adopting a mixed methods approach allowed us to determine the distribution of language preferences within the community, *and* the reasons underlying these preferences. Although given less weight in previous discussions, the latter is crucial since autistic people's language preferences often result from deep reflection on discrimination, ableism, and their identity.

## METHOD

See https://osf.io/t6vqe for the pre‐registration relating to this report.

### Participants

In total, 654 autistic people responded to this survey across 30 different countries (*M*_age_ = 31.90). Participants' gender, diagnostic information, countries of residence, level of education, and races can be seen in Table 1 (see Supplementary Materials A for full ethnicity data). All participants were fluent in English.

**TABLE 1:** Participant demographics

Demographic factor	Group	Number of participants
Gender	Cisgender female	248
	Cisgender male	113
	Transgender female	14
	Transgender male	27
	Non‐binary of third gender	163
	Other	41
	Not disclosed	48
Diagnosis	Autism of Autism Spectrum Disorder	304
	Asperger's syndrome	163
	PDD‐NOS	11
	Other	40
	Self‐identified	128
	Not disclosed	8
Country of residence	Australia	90
	Austria	1
	Belgium	1
	Canada	109
	Denmark	2
	Finland	2
	France	2
	Germany	7
	Greece	1
	Honduras	1
	Hong Kong	2
	Iceland	1
	India	3
	Ireland	85
	Jamaica	3
	Kenya	1
	Luxembourg	1
	Mexico	1
	The Netherlands	6
	New Zealand	54
	Norway	1
	Romania	1
	Slovakia	1
	South Africa	27
	Sweden	2
	Switzerland	1
	United Kingdom	112
	United States of America	135
	Vanuatu	1
Level of education	Primary education	6
	Lower secondary education	28
	Upper secondary education	206
	Post‐secondary non‐tertiary education	29
	Short‐cycle tertiary education	17
	Bachelor's of equivalent level	184
	Master’ s of equivalent level	127
	Doctoral or equivalent level	35
	Not elsewhere classified	4
	Not disclosed	18
Racial group	Asian	27
	Black	8
	Hispanic/Latinx	6
	White	555
	Mixed/Multiple Racial Groups	50
	Not disclosed	8

We recruited participants via an international autism research database (part of the U21 Autism Research Network collaboration), social media advertising (e.g., Twitter, Facebook, Reddit), and through emails to autism charities and organizations across the globe between April 2021 and October 2021. Participants were entered into a prize draw with the chance to win one of multiple £50 (or equivalent local currency) Amazon vouchers (1 in 50 chance of winning). This study was approved by the Science, Technology, Engineering and Mathematics (STEM) ethics committee at the University of Birmingham (ERN_16‐0281AP10) and was conducted in accordance with the principles of the revised Declaration of Helsinki (World Medical Association, 2013).

### Design

This mixed‐methods investigation employed a convergent parallel design (Creswell & Plano Clark, 2017): the quantitative and qualitative elements were conducted in the same phase of the research process, were prioritized equally and analyzed independently, and then interpreted together. Following this design allowed us to identify areas of convergence, divergence, and nuance across the two sources of data (e.g., using ‘autistic’ as a noun; see Results), and understand the reasons underlying participants preferences in that moment (due to the research following a concurrent rather than sequential design).

### Materials and procedure

Participants completed an online survey using the Qualtrics survey platform (https://www.qualtrics.com/). The survey began with a series of demographic questions, including participant age, gender, race, ethnicity, country of birth, country of residence, diagnostic status, type of diagnosis, and level of education (for analyses comparing preferences across genders, and between formally diagnosed and self‐identified autistic individuals, see Supplementary Materials E and F, respectively). Following this, participants completed the autism‐related language preferences survey. For this survey, participants were asked to identify, by selecting from a list, which terms they prefer to use:

- when talking about autism (Asperger's syndrome, Autism, Autism Spectrum Condition, Autism Spectrum Disorder);
- to describe themselves or refer to someone else with autism (Aspie, Autistic, Autistic person, Neurodivergent person, Person on the autism spectrum, Person with autism/ASD/ASC);
- to refer to someone's autistic identity (Has a diagnosis of autism/ASD/ASC/Asperger's, Has Autism/Asperger's, Is autistic/Aspergic, Is neurodivergent);
- when talking about autism more broadly (Condition, Disability, Disease, Disorder, Neurological/Brain Difference);
- to talk about the challenges associated with autism (Challenges, Deficits, Differences, Difficulties, Impairments, Lower/Higher performance, Poorer/Better performance); and
- when talking about people without a diagnosis of autism (Allistic people, Allistics, Control participants, Controls, Healthy Controls, Neurotypical people, Neurotypicals, Non‐autistic people, Non‐autistics, Typical people, Typically developing people^3^).

For each of these terminology categories, participants were asked to select as many terms as they would be *happy to use*, and then select their *favorite* term. In addition, for all questions, participants were given the opportunity to select ‘Other’ and provide an alternative suggestion. For some questions, we included examples to demonstrate the use of the terms in a sentence (e.g., ‘5 autistics participated in this study’) to facilitate comprehension. Finally, in an open question, participants were given the opportunity to tell us more about their language preferences. The survey took approximately 10–30 minutes to complete. The full list of questions, and information on how we confirmed the veracity of survey responses, can be seen in Supplementary Materials B and C, respectively.

### Community involvement

Following participatory research guidelines (Fletcher‐Watson et al., 2019; Keating, 2021), we developed the survey in consultation with several members of the autism community from the Birmingham Psychology Autism Research Team Consultancy Committee. To orient committee members to the topic, author CTK delivered a short presentation summarizing previous literature on this topic, giving suggestions for study aims, and showing a draft of the language preferences survey. The community members identified an additional aim for the study (determining preferences for how we refer to non‐autistic people), highlighted extra autism‐related terminology that should be included (e.g., ‘allistic’, ‘is neurodivergent’, etc.), and provided feedback regarding the length and clarity of questions. These community members were renumerated for their time. In addition, a number of autism charities across the globe were involved in data collection (see Acknowledgements), and there was community involvement during write‐up (author RM).

## RESULTS

### Quantitative results

#### Preferences in the global sample

**FIGURE 1:**
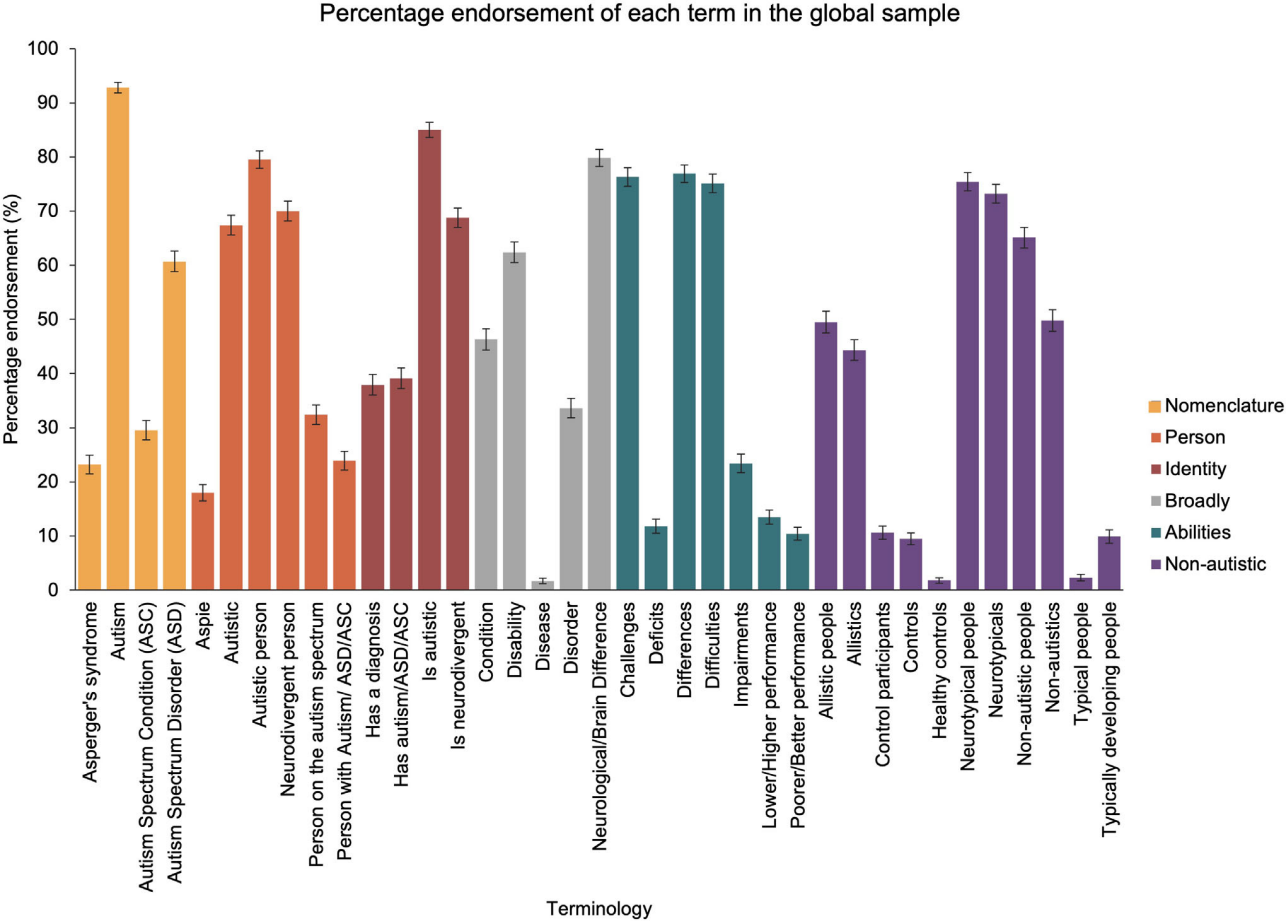
A graph displaying the percentage of participants that endorsed each of the terms within each category

For the following analyses, the data for all participants were amalgamated into one group to explore language preferences in a large and diverse sample. To explore the percentage of participants who would be ‘happy to use’ our autism‐related terms (see Supplementary Materials D for results relating to favorite terms), we conducted 6 one‐way ANOVAs. In the first ANOVA, we assessed language preferences relating to the nomenclature of autism. This identified a significant main effect [*F*(2.78, 1817.61) = 407.97, *p* < 0.001, *η*_P_^2^ = 0.39]: the term that was endorsed by the highest percentage of participants was ‘Autism’ [92.8%], followed by ‘Autism Spectrum Disorder’ [60.7%], followed by ‘Autism Spectrum Condition’ [29.5%], and then ‘Asperger's syndrome’ [23.2%; see Figure 1]. The second ANOVA assessed preferences relating to the self/person. This revealed a significant main effect [*F*(4.10, 2677.13) = 260.03, *p* < 0.001, *η*_P_^2^ = 0.29]: the term that was endorsed by the highest proportion of the participants was ‘Autistic person’ [79.5%], followed by ‘Neurodivergent person’ [70.0%] and ‘Autistic’ [67.4%], followed by ‘Person on the spectrum’ [32.4%], and finally ‘Person with Autism/Autism Spectrum Disorder/Autism Spectrum Condition’ [23.9%], and Aspie [18.0%; see Figure 1]. In the third ANOVA, we assessed language preferences for referring to someone's autistic identity. This found a significant main effect [*F*(2.73, 1783.54) = 182.25, *p* < 0.001, *η*_P_^2^ = 0.22]: ‘Is autistic’ was endorsed by the highest percentage of participants [85.0%], followed by ‘Is neurodivergent’ [68.8%], followed by ‘Has Autism/Autism Spectrum Disorder/Autism Spectrum Condition’ [39.1%] and ‘Has a diagnosis of Autism/Autism Spectrum Disorder/Autism Spectrum Condition’ [37.9%; see Figure 1]. The fourth ANOVA, which assessed language preferences relating to how autism is conceptualized more broadly, identified a main effect [*F*(3.50, 2286.41) = 348.37, *p* < 0.001, *η*_P_^2^ = 0.35]. The term that was endorsed by the highest percentage of participants was ‘Neurological/Brain Difference’ [79.8%], followed by ‘Disability’ [62.4%], followed by ‘Condition’ [46.3%], followed by ‘Disorder’ [33.6%], and finally ‘Disease’ [1.7%; see Figure 1]. In the fifth ANOVA, we assessed language preferences relating to how we talk about the difficulties of autistic people. This identified a significant main effect [*F*(5.09, 3322.35) = 563.43, *p* < 0.001, *η*_P_^2^ = 0.46], revealing that ‘Differences’ [76.9%], ‘Challenges’ [76.3%] and ‘Difficulties’ [75.1%] were endorsed by the highest percentage of participants, followed by ‘Impairments’ [23.4%], followed by ‘Lower/Higher Performance’ [13.5%], ‘Deficits’ [11.8%] and ‘Poorer/Better Performance’ [10.4%; see Figure 1]. Finally, in the sixth ANOVA, which assessed preferences relating to how we refer to non‐autistic people, we found a significant main effect [*F*(6.18, 4032.37) = 431.01 *p* < 0.001, *η*_P_^2^ = 0.40]. The terms that were endorsed by the highest percentage of participants were ‘Neurotypical people’ [75.4%] and ‘Neurotypicals’ [73.2%], followed by ‘Non‐autistic people’ [65.1%], followed by ‘Non‐autistics’ [49.8%] and ‘Allistic people’ [49.5%], followed by ‘Allistics’ [44.3%], followed by ‘Control participants’ [10.6%], ‘Typically developing people’ [9.9%], and ‘Controls’ [9.5%], and then finally ‘Typical people’ [2.3%] and ‘Healthy Controls’ [1.8%] (see Figure 1).

**FIGURE 2:**
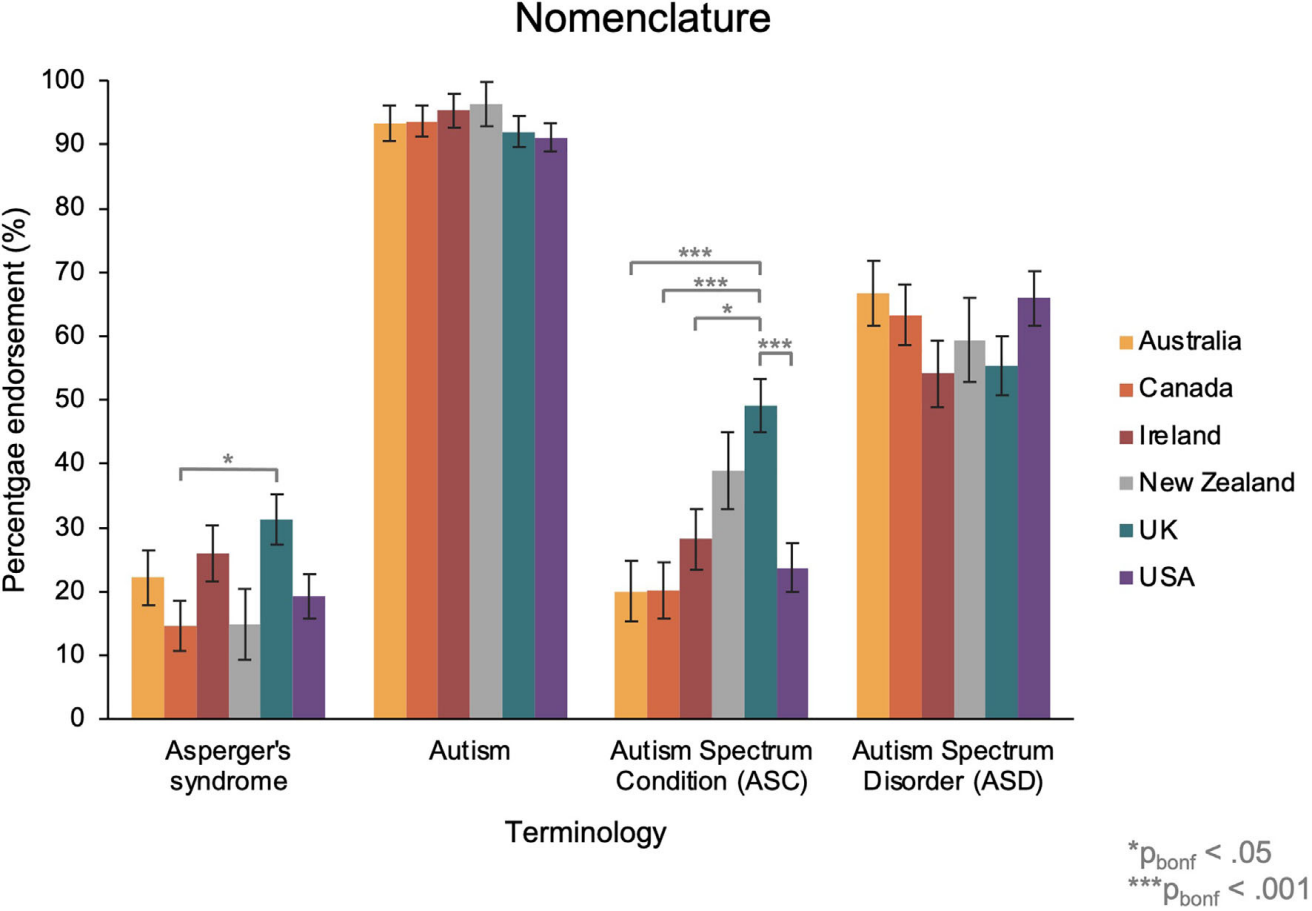
The percentage of participants in each country group that endorsed the terms relating to the nomenclature of autism. Australia in yellow, Canada in orange, Ireland in red, New Zealand in gray, United Kingdom in blue and United States in purple

#### Comparing autism‐related language preferences across countries

As per our pre‐registration (see https://osf.io/t6vqe), for our analyses comparing language preferences across countries, we only included the data from country groups that had at least 50 participants (in which we had sufficient data to draw comparisons between groups). We conducted six mixed ANOVAs, with the between‐subjects factor *country* (Australia, Canada, Ireland, New Zealand, United Kingdom, United States of America), and the within‐subjects factor *term*, to assess the proportion of participants that would be ‘happy to use’ each of the terms across countries (see Supplementary Materials D for results relating to favorite terms). In the first ANOVA, which assessed language preferences relating to the nomenclature of autism, we identified a significant main effect of *term* (as expected from the analysis above) [*F*(2.77, 1605.48) = 365.94, *p* < 0.001, *η*_P_^2^ = 0.39], and a significant term × country interaction [*F*(13.86, 1605.48) = 3.63, *p* < 0.001, *η*_P_^2^ = 0.03]. Bonferroni‐corrected pairwise comparisons demonstrated that a higher percentage of participants endorsed the term ‘Asperger's syndrome’ in the United Kingdom [31.3%] than in Canada [14.7%, *t*(207.35) = 2.98, *p*_bonf_ = 0.042]; and a higher percentage of participants endorsed term ‘Autism Spectrum Condition’ in the United Kingdom [49.1%] than in Australia [20.0%, *t*(200) = 4.57, *p*_bonf_ < 0.001], Canada [20.2%, *t*(211.42) = 4.73, *p*_bonf_ < 0.001], Ireland [28.2%, *t*(189.23) = 3.06, *p*_bonf_ = 0.017], and the United States of America [23.7%, *t*(218.83) = 4.23, *p*_bonf_ < 0.001]. There were no other significant differences (after Bonferroni‐correction) in percentage endorsement of terms between countries (see Figure 2).

**FIGURE 3:**
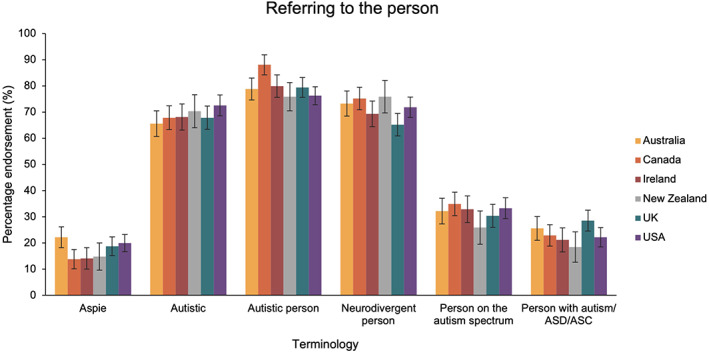
The percentage of participants in each country group that endorsed terms for referring to an autistic person. Australia in yellow, Canada in orange, Ireland in red, New Zealand in gray, United Kingdom in blue and United States in purple

The second ANOVA compared preferences relating to the self/person across countries. This revealed that there was a significant main effect of term [*F*(4.12, 2384.34) = 233.01, *p* < 0.001, *η*_P_^2^ = 0.29], but no term x country interaction [*F*(20.59, 2384.34) = 0.71 *p* = 0.83, *η*_P_^2^ = 0.01; see Figure 3].

**FIGURE 4:**
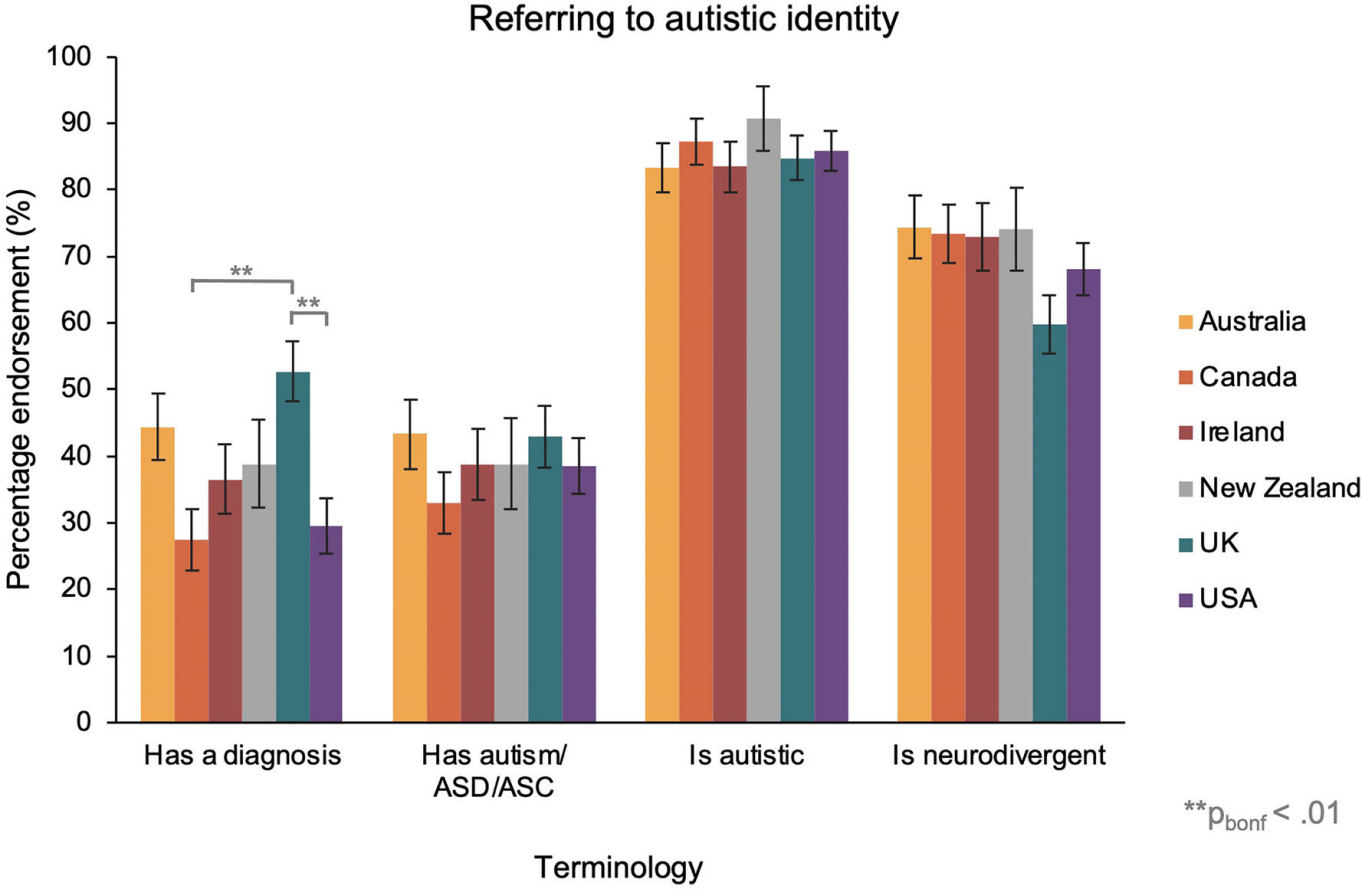
The percentage of participants in each country group that endorsed terms for referring to someone's autistic identity. Australia in yellow, Canada in orange, Ireland in red, New Zealand in gray, United Kingdom in blue and United States in purple

In the third ANOVA, we compared language preferences for referring to someone with autism. This found a significant main effect of term [*F*(2.71, 1569.25) = 162.29, *p* < 0.001, *η*_P_^2^ = 0.22], and a term x country interaction [*F*(13.55, 1569.25) = 2.24, *p* = 0.006, *η*_P_^2^ = 0.02]. Bonferroni‐corrected pairwise comparisons revealed that a higher percentage of participants endorsed the term ‘Has a diagnosis of autism/ASC/ASD’ in the United Kingdom [52.7%] than in Canada [27.5%, *t*(217.48) = 3.93, *p*_bonf_ = 0.002] and the United States [29.6%; *t*(227.60) = 3.74, *p*_bonf_ = 0.003]. There were no other significant differences in percentage endorsement between countries (see Figure 4).

**FIGURE 5:**
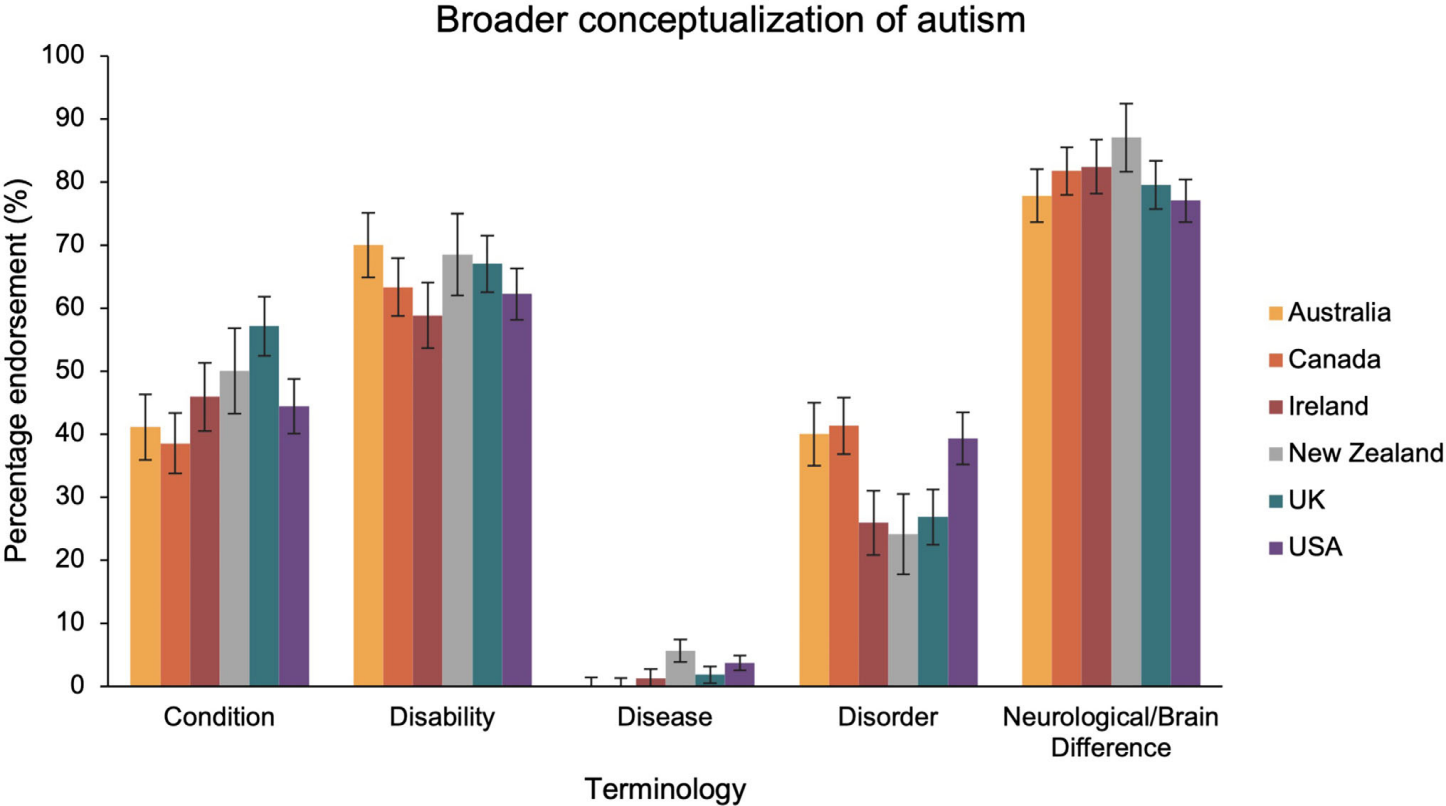
The percentage of participants in each country group that endorsed the terms for conceptualizing autism more broadly. Australia in yellow, Canada in orange, Ireland in red, New Zealand in gray, United Kingdom in blue and United States in purple

The fourth ANOVA, which compared the language preferences relating to how autism is conceptualized more broadly across countries, identified a main effect of term [*F*(3.52, 2037.48) = 304.17, *p* < 0.001, *η*_P_^2^ = 0.34] and a term x country interaction [*F*(17.60, 2037.48) = 1.95, *p* = 0.010, *η*_P_^2^ = 0.02]. Whilst there was clear variation in the percentage of participants that endorsed each of the five terms across country groups (see Figure 5), there were no statistically significant differences after Bonferroni‐correction [*p*_bonf_ > 0.05].

**FIGURE 6:**
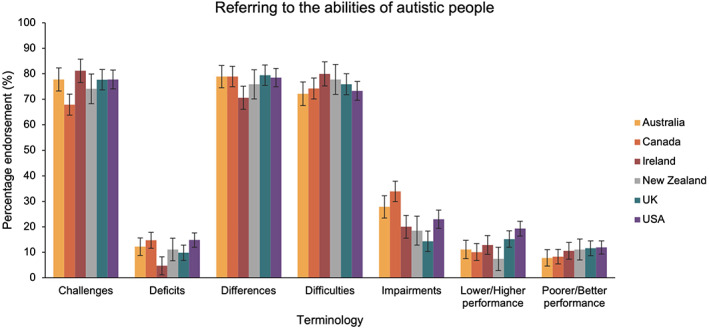
The percentage of participants in each country group that endorsed terms for referring to the difficulties of autistic people. Australia in yellow, Canada in orange, Ireland in red, New Zealand in gray, United Kingdom in blue and United States in purple

In the fifth ANOVA, we compared language preferences relating to how we talk about the difficulties of autistic people across country groups. This identified a significant main effect of term [*F*(5.10, 2954.34) = 472.80, *p* < 0.001, *η*_P_^2^ = 0.45], and a term x country interaction [*F*(25.51, 2954.34) = 1.52, *p* = 0.046, *η*_P_^2^ = 0.01]. Whilst there was clear variation in the percentage of participants that endorsed each of the seven terms across country groups (see Figure 6), there were no statistically significant differences [*p*_bonf_ > 0.05].

**FIGURE 7:**
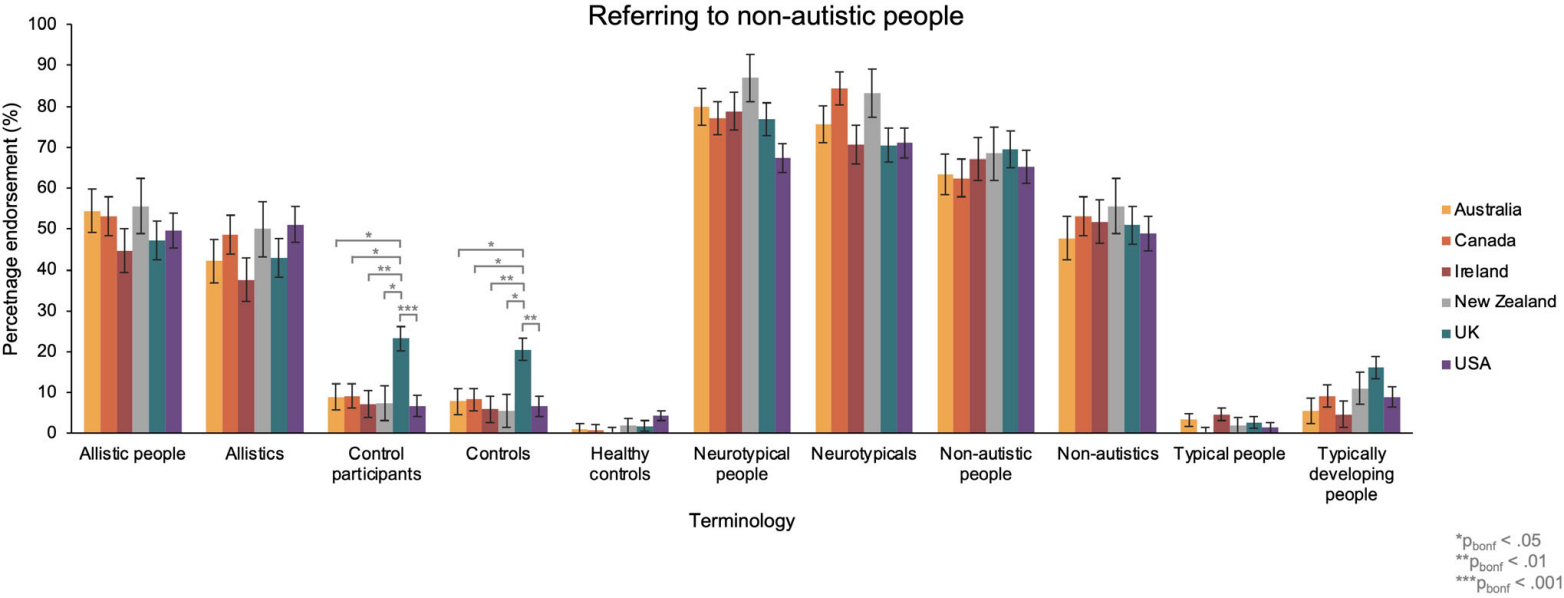
The percentage of participants in each country group that endorsed terms for referring to non‐autistic people. Australia in yellow, Canada in orange, Ireland in red, New Zealand in gray, United Kingdom in blue and United States in purple

Finally, in the sixth ANOVA, which compared preferences relating to how we refer to non‐autistic people across countries, we found a significant main effect [*F*(6.15, 3562.32) = 388.62, *p* < 0.001, *η*_P_^2^ = 0.40], and a term x country interaction [*F*(30.76, 3562.32) = 1.52, *p* = 0.033, *η*_P_^2^ = 0.01]. Bonferroni‐adjusted pairwise comparisons demonstrated that a higher percentage of participants endorsed the terms ‘Control participants’ [23.2%] and ‘Controls’ [20.5%] in the United Kingdom than all other countries studied [‘Control participants’: 6.7%–9.2%; ‘Controls’: 5.6%–8.3%; all *p*_bonf_ < 0.05]. There were no other significant differences in percentage endorsement of terms between countries (see Figure 7).

**FIGURE 8:**
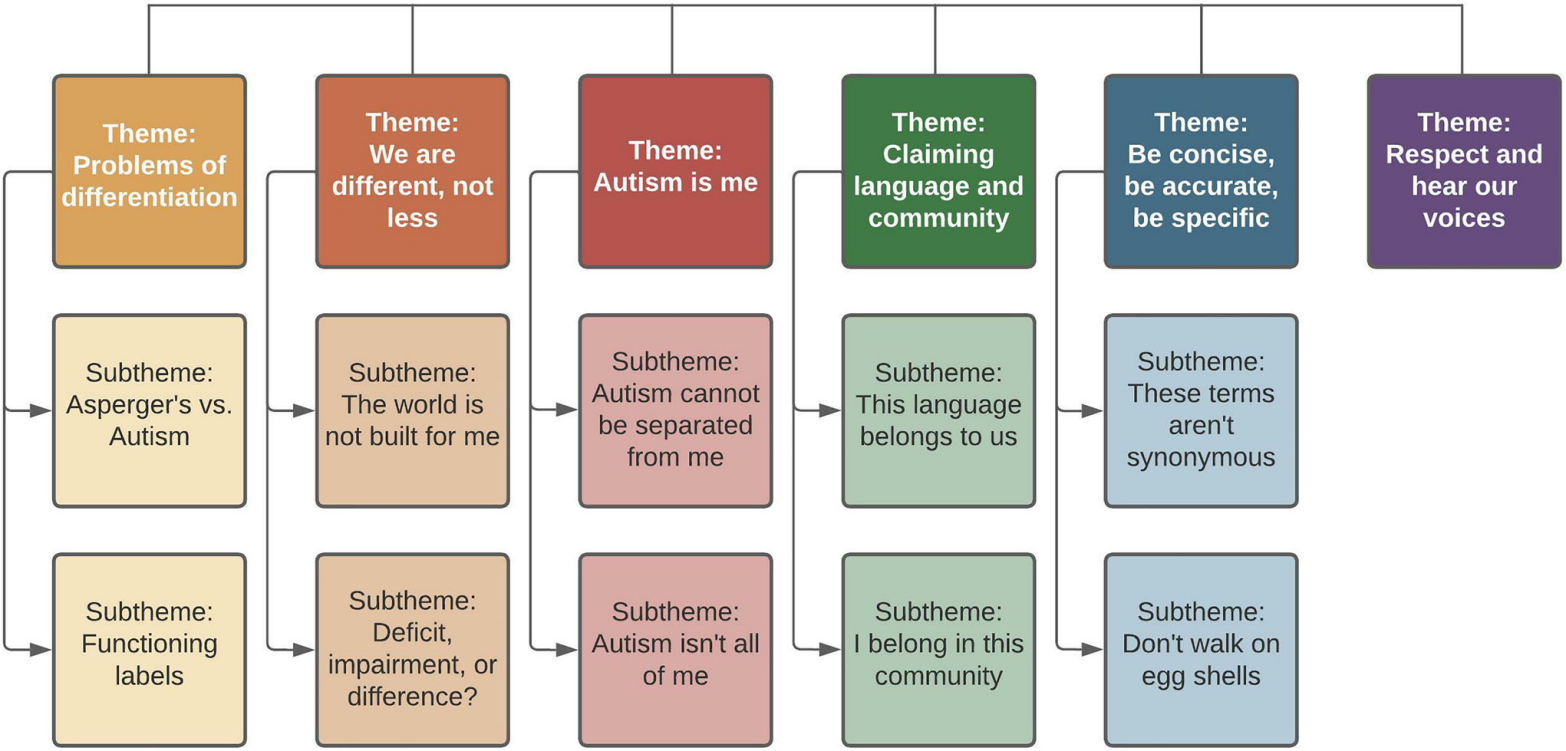
A diagram depicting the thematic structure arising from participants' responses

### Qualitative results

There were 414 responses to the open question which asked participants to tell us more about their autism‐related language preferences (see Supplementary Materials G for the number of respondents per country). As the aim of the qualitative arm of this project was to understand the variation in language preferences, and to centralize the voices of the community, we conducted a Reflexive Thematic Analysis of the responses (Braun & Clarke, 2019). Thematic analysis was particularly suitable for this project as it is sensitive to exploring nuanced meanings within data (Braun & Clarke, 2006, 2019; King, 2004), is amenable to larger datasets (Braun & Clarke, 2006), and allows exploration of diverse perspectives (Nowell et al., 2017).

We (CTK and HH) followed the steps of Braun and Clarke (2006, 2019) for conducting a Reflexive Thematic Analysis, adopting an inductive approach within a contextualist framework, wherein the social context was considered to shape participants' accounts of their preferences (Braun & Clarke, 2013). Both authors read all responses to centralize the voices of the participants within the analytic process and to familiarize themselves with the data. Following this, the authors independently coded the transcripts and met at regular intervals (25% of responses, 50% of responses, 100% of responses) to discuss initial codes, identify cross‐over, and resolve discrepancies. Once coding was completed, the authors discussed the thematic structure of the coding, and generated initial themes representing consistent patterns within the data. During this stage, example quotes were highlighted, efforts were made to ensure the nuances of the data were represented in the thematic overviews, and deviant cases were identified and integrated. Finally, the themes were reviewed for relevance, internal consistency and external distinctiveness, and hierarchies within the themes were explored through the generation of subthemes which represented further patterns of meaning within the data (thematic structure can be seen in Figure 8).

### Thematic analysis

Overarching the data was a clear message from the respondents that they wanted greater recognition and centralizing of their voices within policy, research, and everyday parlance. This is explored in more detail through the six core themes, *Problems of differentiation; We are different, not less; Autism is me; Claiming language and community; Be concise, be accurate, be specific; and Respect and hear our voices*.

#### Theme: Problems of differentiation

*Problems of differentiation* addresses participants' stance on terms used to segregate autistic people (i.e., functioning labels and ‘Asperger's syndrome’) as being inaccurate, harmful and divisive, and having a problematic history. This theme can be separated into two subthemes, *Asperger's* vs. *Autism*, and *Functioning labels*.

I dislike Asperger's as a term, not just because of the troubled history of the namesake, but also because I believe it segregates us. The point of Asperger's originally was to pin us down into 'higher' and 'lower' functioning labels and I massively object to that. I would be labelled as classically 'high functioning'… but just because I might have less support needs than other Autistic people doesn't mean I don't have days where my support needs are quite high. It fluctuates by day, week, month. So to pin people down into labels of how well they 'function' is unhelpful and irritates me.’ (Participant 170, Ireland, pg. 19, lines 16‐23)

Participants identified that ‘Asperger’ and functioning labels unnecessarily ‘segregate’ people that are all part of the same spectrum. Citing variation in functioning across time and situations they felt that this differentiation was unhelpful for autistic people, resulting in poorer support, understanding, and agency for both ‘high‐functioning’ and ‘low‐functioning’ individuals.

#### Subtheme: Asperger's versus autism

Participants reported that the term ‘Asperger's’ is harmful and has connotations with a eugenical Nazi regime.

Aspergers Syndrome in particular was a term coined by Hans Asperger, an actual, literal Nazi doctor and eugenicist. The distinction between people with "Aspergers Syndrome" and other autistic people, in his view, was that people with Aspergers could work and assimilate into neurotypical society reasonably well (and therefore deserved to live), and autistic people could not (and therefore deserved to die). (Participant 92, Canada, pg. 10, lines 41‐45)

A large proportion of participants asserted that ‘Asperger’ should not be used due to its links to Hans Asperger‐ a eugenicist Nazi doctor. The term ‘Asperger’ was identified as a means by which to separate autistic individuals based on perceived utility for society. Consequently, the participants stated that this inappropriately validated the use of functioning level descriptors and endorsed distinguishing people based on an outsider's perspective of their social usefulness.

Despite respondents eschewing the term, several respondents highlighted that ‘Asperger’ is used by some autistic people as a means of separating themselves from ‘inferior’ autistic people, termed ‘Aspie supremacy’.

I have found that when people cling to the term Aspie it's either because this was their original diagnosis (and I do get why that would lead to an affinity for the term) or because they want to see themselves as being 'better than those /really/ autistic people over there' (sometimes called 'Aspie supremacy'). This is noxious. (Participant 151, Ireland, pg. 17, lines 8‐12)

Respondents identified several reasons why some preferred using the term ‘Asperger's’. For example, respondents felt others were using the term because it was a core part of their identity following diagnosis, provided perceived protection from the ‘stigma associated with autism’, or made them feel ‘part of an elite club’ or ‘superior’ to other autistic people. Participants were particularly concerned with the latter manifestation as it was entrenched in ableism, suggesting that those with higher support needs are ‘inferior’ to those with Asperger's. This is also reflected in the accounts of respondents who liked to refer to themselves as ‘Aspie’ or as having ‘Asperger's’, though, rather than being framed around ‘superiority’, their preference was framed in ways that highlighted their different support needs and a wish to reflect their initial diagnosis.

#### Subtheme: Functioning labels

Autistic participants felt that *Functioning label* descriptors were inaccurate, harmful, and divisive. The respondents highlighted that since functioning varies across time and situations, the descriptors ‘low‐functioning’/‘high‐functioning’, and ‘mild’/'severe’ were reductionist and inaccurate.

The terms "High/Low functioning" are terrible and make the spectrum sound like I would be high and my brother (also autistic, 28 years of age) would be low, but to counter that, my brother doesn't know he is autistic because he does not understand that, but tries to live his life as if he wasn't different. For example, he once held a job, whereas I couldn't/can't at all. He is a social butterfly, whereas I shut down and panic in social situations. I could drive, he can't. He wants to be a father and husband, I do not wish to have a partner or children AT ALL. The idea that you are either high or low is immediately debunked by my brother and I. (Participant 105, Canada, pg. 12, lines 14‐21)

Functioning descriptors were considered to be inaccurate as ‘functioning’ fluctuates across time and situations. For the participants, functioning labels represented inflexible structures and conceptions of autism, and diminished the nuances of functioning capabilities.

In reducing ‘functioning’ to simplistic and categorical grouping of high‐ or low‐functioning, participants identified that support needs and agency of autistic people were often ignored, and consequently autistic people suffered.

Because I am seen as 'high‐functioning,' it means people are less likely to give me accommodations that I need to be successful. They believe I should just cope with things (e.g. bright lights/loud noises/busy environments) like they do. Likewise, when people are labelled 'low‐functioning' (maybe they don't use their voice to communicate or they need help with basic tasks), their strengths and abilities are minimized. They aren't allowed to advocate for themselves and they are denied basic agency. We are all *just autistic.* We are all completely different from each other. Grouping us by how outsiders perceive our level of ability is inaccurate and harmful. (Participant 151, Ireland, pg. 17, lines 21‐28)

Functioning labels were considered unhelpful and harmful for autistic individuals. As Participant 151 highlights, the use of functioning labels means that the support needs of ‘high‐functioning’ individuals may be overlooked or underestimated, and ‘low‐functioning’ individuals may have their strengths or abilities minimized and be denied agency or voice.

The application of functioning labels was also felt to be a consequence of how ‘outsiders’ (i.e., non‐autistic people) perceived their level of ability or contribution to society.

This evaluation of functioning is based solely on capitalistic definitions of worth defined by neurotypicals, and labeling autistic people only by how much they can contribute to a capitalist society can do damage to their self esteem. (Participant 77, pg. 9, Canada, lines 27‐30)

Participants felt that rather than the autistic person's perception of their abilities leading their support needs, they were instead subject to functioning labels based on neurotypical definitions of ‘worth’ and contributions to society. Consequently, the labels applied to them often represented a mismatch in perceptions of functioning, leading to lower self‐esteem. Some participants suggested that functioning labels were not there to help them, but instead were used to help neurotypical people better understand the diagnosis and support needs.

#### Theme: We are different, not less

Participants viewed deficit‐based language as harmful and inaccurate, with many of these so‐called ‘deficits’ arising as a result of being placed within a world not built for autistic people, rather than due to any inherent deficiency within themselves.

My brain formed a bit differently and that doesn't make me sick or disordered. It only appears this way because of the pressures and expectations placed on individuals by our current societal model. (Participant 158, Ireland, pg. 18, lines 5‐7)

Respondents routinely felt that they were positioned as being inferior to non‐autistic people, and critiqued language that positioned them as ‘a burden’, ‘sick’ or ‘disordered’. Instead, participants preferred autism to be considered, taking a more critical stance to the neurotypical ‘societal model’, as ‘a difference in brain‐wiring’, and that these differences should be recognized without judgment. This theme can be separated into two subthemes, *Deficit, impairment or difference*, and *The world is not built for me*.

#### Subtheme: The world is not built for me

Participants reported that a large proportion of the difficulties they face are caused by living in a neurotypical society, rather than the difficulties being inherent to them.

Most "challenges", "difficulties" and "problems" only arise when I as an autistic person have to communicate with a world designed by and for neurotypical people. (Participant 140, Germany, pg. 15, lines 25‐27)

I don't believe that autism is an inherent disability, only that we're disabled by the society we live in. (Participant 227, New Zealand, pg. 25, lines 45‐46)

It is a neurological difference which causes difficulties in a neurotypical world that is all (Participant 312, United Kingdom, pg. 39, lines 25‐26)

Try to imagine what a world made for autistic people would look like. I guarantee you we‘d be absolutely fine (Participant 59, Austria, pg. 7, lines 42‐43)

In line with the social model of disability (Shakespeare, 2006), many respondents noted that their ‘difficulties’ arose from being situated in a neurotypical world not built for them. Consequently, participants felt that *society* was disabling for autistic people and if the world *was* built for them, with accommodations made to their differences and support needs, then many of their difficulties, or even all of them, would dissipate.

Whilst there was a recognition of the problematic structure of an ableist society, participants still noted that for some autistic individuals legitimate difficulties may remain even after accommodations.

I think there is no shame in… acknowledging that autism is also a disability, both in social model terms, i.e. that often we are disabled by it because of society but also that there are inherent aspects of being autistic that disable us that an ideal society wouldn't erase (Participant 307, United Kingdom, pg. 36, lines 41‐44).

I have a disability but not every autistic person is disabled by their autism. Then I would say that for them it's merely a neurological difference (Participant 60, Belgium, pg. 8, lines 3‐4).

From the responses, we can see awareness of the fact that environments and reasonable adjustments having different impacts on different autistic people; for some difficulties may disappear when their differences are accommodated (e.g., turning lights down in shops due to photosensitivity), but for others these difficulties (e.g., ‘find[ing] it hard to break a routine’) may remain after reasonable adjustments. Therefore, we must recognize that each individual will experience autism in different ways, and acknowledge that support needs vary from person to person, across different situations, and over time.

#### Subtheme: Deficit, impairment or difference?

Participants held a nuanced stance on the use of terms like *Deficit, impairment or difference*, situating appropriateness in relation to what the terms are being used for.

Language vis a vis "impairments" should be dependent on what it's being used for. For example, my SPD is an impairment or disability, but not giving people eye contact is just a difference. My stutter is more an annoyance than a real impairment, for me. It really depends on whether it's the internal thing that's preventing people from reaching their goals, or whether it's external pressures on them which are more disabling. For example, the eye contact thing means that I am occasionally misconstrued as being impolite, but that's entirely because of cultural assumptions made by the other person (eye contact is not actually appropriate in every culture), but the SPD must be managed regardless of other people's reactions. (Participant 18, Australia, pg. 2‐3, lines 46‐7)

Participants stipulated that language use should depend on where the ‘difficulty’ comes from. For example, it should depend on whether there is something internal that is preventing people from reaching their goals, or external pressures which are disabling. To illustrate, Participant 18 highlights that whilst their sensory processing disorder (SPD) can be seen as an ‘impairment’ or ‘disability’ as it is something that requires support, their stutter and levels of eye contact are just ‘differences’.

In addition, some respondents noted that pathologizing language is even used to refer to traits that are diagnostically relevant rather than any intrinsic ‘deficit’.

I do think a lot of autistic personality traits are needlessly pathologized because they're useful indicators for diagnosis, but just because it's diagnostically relevant doesn't mean it's an impairment (Participant 412, United States of America, pg. 53, lines 1‐3)

This participant noted that some autistic traits that are diagnostically relevant are needlessly pathologized. This report, along with many others, demonstrates that there is a tendency to pathologize autistic characteristics and behavior (e.g., stimming, differences in eye contact, etc.), even when these stem from living in a neurotypical society (rather than autism itself), or do not confer any detriment to autistic people themselves.

#### Theme: Autism is me

Participants repeatedly emphasized that autism is an integral part of who they are and that it cannot, and should not, be separated from them.

Autistic is me, my personality, my imagination, my soul, my spirit. If autism disappeared so would I. (Participant 189, United Kingdom, pg. 21, lines 21‐22)

Participants consistently stated that being autistic was an elemental or large part of their identity and should be acknowledged as such. Typically, these kinds of responses were coupled with criticism of person‐first language, which implies that autism is separate to them. This theme has two subthemes, *Autism cannot be separated from me*, and *Autism is not all of me*.

#### Subtheme: Autism cannot be separated from me

*Autism cannot be separated from me*, encompasses participants' opinion that autism cannot and should not be separated from them.

I dislike "person with autism" or "person that has autism" because it's a diagnosis, not a physical item/possession. Items and possessions can always be put down or put away. I can't just "put my autism down" and suddenly not be autistic. It's not a handbag. (Participant 12, Australia, pg. 2, lines 12‐15)

A multitude of participants critiqued person‐first language, noting that autism is not ‘an accessory’, ‘possession’, or ‘add‐on’. Instead, participants supported the use of identity‐first language which reflected the elemental and indelible role that autism played in their identity.

Participants further disliked person‐first language as it tapped into eliminationist and ableist positions on autism.

Using terms like "person with autism" feels like an attempt to separate it from me as if it were a disease, and these terms are commonly used by groups of people who ignore autistic voices and support things like a 'cure' for autism. (Participant 130, Canada, 14, 19–22)

Synonymous with diseases (e.g., ‘person with cancer’), person‐first language was considered inappropriate for use with autism, and participants raised concerns that this type of language (which suggests autism could be separated from them) incentivizes attempts to find a ‘cure’ as if it were a disease to be eradicated. Instead, participants advocated the use of identity‐first language, and drew on comparisons with race and sexuality to emphasize how linguistically inappropriate and illogical person‐first language is (e.g., ‘a gay person doesn't ‘have homosexuality’ they are just gay’).

#### Subtheme: Autism is not all of me

Whilst participants wanted their autism to be recognized as integral part of them, many also did not want autism to be considered as all that they are, and they highlighted the importance of acknowledging personhood.

I don't like the term "autistic" as a noun though. I'm not an autistic. I'm a person… I'm more than [an] autistic. I'm human. (Participant 123, Canada, pg. 13, lines 42‐45)

Many participants highlighted that using ‘autistic’ as a noun (i.e., ‘an autistic’ or ‘that group of autistics’) was reductive and dismissive of their personhood. These participants typically noted that they preferred identity‐first language and emphasized that using ‘autistic’ as a noun is dehumanizing and derogatory.^4^

I do generally try to avoid noun omission and use terms like "Autistic people" and "autists" over "autistic" as a noun as this omission of noun is often used to subtlety dehumanize marginalized groups (eg "blacks" vs "black people’…). (Participant 409, United States of America, pg. 52, lines 25‐28)

As demonstrated by participant 409, respondents noted that noun omission was ‘often used to subtly dehumanize marginalized groups’. Respondents drew comparisons with other marginalized groups (e.g., ‘blacks vs. black people’; ‘gays’ vs. ‘gay people’) to emphasize how problematic noun‐use is. Specifically, participants wanted autism to be recognized as a key part of who they are without reducing them to solely that part of them or being dehumanized.

#### Theme: Claiming language and community

Activism was a substantial driver of language preference within the autistic community. This activism could be split into two broad categories which is reflected in our two subthemes *This language belongs to us* and *I belong in this community*.

Language I do like is language that empowers us and allows us to self‐identify (the word 'autistic' does this), language that acknowledges our autism as being an integral part of us, language that allows us to express that we are different, not less, and most importantly, language that the autistic community develops ourselves instead of words that are forced onto us by non‐autistic people (Participant 42, Australia, p. 5, lines 41‐45)

Respondents often spoke of a strong connection to the autism community which informed their language preferences. Some felt that language underpinned appropriate support and treatment by society, researchers, and health professionals, and their choice of language was a political and community‐spirited act. Other respondents, however, stated that tone, intent, and respect were more important than the language itself.

Personally I do not care which words are used to describe me as long as I know the person has good intentions and is not trying to be offensive. (Participant 97, Canada, pg. 11, lines 11‐12)

#### Subtheme: This language belongs to us

Reclamation of language was shown by many respondents, and they specified who could and could not use certain terms.

I do not like it when neurotypical people call a group of autistic people ‘autistics’, as this gives them the option to dehumanize us. I believe only autistic/other neurodivergent people should say this. (Participant 81, Canada, pg. 9, lines 41‐43)

Respondents reclaimed ‘an autistic’ for those within their community and argued non‐autistic people who used it were tapping into dehumanizing language. In this example, they lean into their community and language, however, participants also leaned into communities outside of the autistic community.

Participants also reclaimed the term ‘disability’ in order to permit access to reasonable adjustments, and to give participants a sense of broader community.

I like using the term disability as it can be used to get accessibility under the equality act, since autism is a disability and it is illegal to discriminate against people based on disability. It has helped me a lot when getting accommodations at university and when trying to make education and healthcare more accessible. It also links [us] to the disabled community which is much larger than the autistic community so we get access to a lot more information and support. (Participant 309, United Kingdom, pg. 38, lines 29‐34)

As participant 309 highlights, the term disability was utilized as a legal tool for gaining access to accommodations in education and healthcare and this was implemented when they felt accommodations were not being met. In doing so, the participants also felt that using disability‐based language permitted greater access to a much larger community with more information and support.

#### Subtheme: I belong in this community

Many respondents also reported using broader‐encompassing terms like ‘neurodivergent’ in order to be part of, and stand in solidarity with, a wider community.

I like the term neurodivergent as since we often have comorbid disorders it allows us to have access to a larger community to learn from and share ideas for accessibility, policy and support (Participant 309, United Kingdom, pg. 37, lines 33‐35)

Using broader terms like ‘neurodivergent’ provided access to a larger community with knowledge of accessibility, policy, and support. Other participants used broader terms like neurodivergent for ‘perceived protection’ and to avoid ‘outing someone' or themselves as autistic.

Many also commented that they did not want to lose their autistic identity and emphasized that recognizing their experience as an autistic person, specifically, is important too.

I generally prefer terms like neurodivergent as dx [diagnosis] boundaries can be fuzzy and I feel that solidarity between different types of neurodiversity and recognizing overlap is important. However there are times when referencing Autistic experience specifically is important, in which case I tend to use Autistic when referring to a specific experience of being dx or identifying as Autistic. (Participant 108, Canada, pg. 12, lines 25‐29)

Whilst recognizing overlap between conditions is important and provided them with greater support and information, participants also wanted to retain their identity as an autistic person, and reported it being crucial to recognize the ‘autistic experience specifically’. In scenarios where participants want people to acknowledge their specific experiences as an autistic person, they use autism‐specific language (e.g., ‘autistic person’ rather than ‘neurodivergent person’).

#### Theme: Be concise, be accurate, be specific

For many respondents, it was important to use language that was concise, accurate, and specific.

Just say what you mean and mean what you say. Accuracy is a form of respect and respect is the currency of human interaction. (Participant 161, Ireland, pg. 18, lines 21‐22)

Here it was emphasized that using accurate language is a form of respect in itself; by using the most appropriate language, you are being respectful too. This theme can be broken down into the two subthemes, *These terms aren't synonymous*, and *Do not walk on egg shells*.

#### Subtheme: These terms are not synonymous

Many respondents wanted specificity in language use. Indeed, they highlighted that the terms ‘neurodivergent’ and ‘neurotypical’ are not synonymous with ‘autistic’ and ‘non‐autistic’, and therefore should not be used interchangeably.

Neurodivergent is not specific to autism, so should not be used as a synonym. Neurotypical should only be used to describe someone who is not neurodivergent; there are neurodivergent people who are not Autistic. (Participant 71, Canada, pg. 9, lines 1‐3)

The participants noted that these terms should not be used interchangeably as ‘neurodivergent’ encompasses a range of other diagnoses such as ADHD and dyslexia, and therefore is not specific to autism. Similarly, the word ‘neurotypical’ rules out other forms of neurodivergence (e.g., ADHD, dyslexia, Tourette's syndrome), whereas non‐autistic does not; it is possible to be non‐autistic but not neurotypical (e.g., if you have ADHD but not autism). This distinction was important to the autistic respondents for the sake of accuracy, and to ensure that they do not lose their specific identity as an autistic person.

#### Subtheme: Do not walk on egg‐shells

Some respondents disliked others trying to be overly polite or avoiding certain language (e.g., autistic) as this often led to less accurate and specific language use.

I generally prefer terms which are specific and don't beat around the bush in order to be polite (Participant 395, United States of America, pg. 50, lines 39‐40)

It is more hurtful when I feel like people are walking on eggshells around me and scared of accidentally offending me, or assuming I'm going to be hypercritical of their language and cry if they use the wrong word. This could lead to me not being included in activities or social circles. (Participant 97, Canada, pg. 11, lines 12‐15)

Several respondents highlighted that we should ‘stop beating around the bush’ or ‘walking on eggshells’ when speaking about autism. Similarly, many participants highlighted that they dislike when people avoid using certain words like ‘disabled’ or ‘disability’ as these are ‘positive political identities’. To avoid saying or doing the wrong thing, respondents felt that others disengage or avoid autistic people, leading to a greater sense of isolation.

#### Theme: Respect and hear our voices

One of the most important themes that we identified centres on the necessity to respect and ask for personal preferences, rather than to assume we know which terms to use.

Even if you know the correct terminology, ask. We are not a monolith. (Participant 128, Canada, pg. 14, lines 14)

Respondents repeatedly noted the varying experiences of autism *and* varying views on language. Consequently, they emphasized the importance of asking autistic people about their language preferences. Many of the autistic participants were mindful that their preferences might not be the same as others' and outlined that they would be happy to use less‐favored terms to respect personal preferences.

If another autistic person personally prefers person first language I will regard them with such because it is their experience with autism to respect, not mine, I just hope they do the same in return when I ask to be called simply, autistic. (Participant 373, United States of America, pg. 48, lines 36‐39)

As demonstrated by Participant 373, respondents were often flexible to the preferences of others, and would use terms that they would otherwise not use to describe themselves. This was often situated within the hope of mutual respect, that their approach to respecting personal preferences would be reciprocated by other autistic people. Despite the preference for mutual respect, this was a point of contention as several respondents noted that some other autistic people did not respect their preferences and imposed upon them the language to use.

The pressure to go with the majority preference of autistic I find as oppressive as when neurotypicals talk about autism, except it is worse because it comes from within the autism community. Why don't they accept difference, including in how someone with autism/autisic person might prefer to self‐identify. (Participant 279, United Kingdom, pg. 32, lines 9‐13)

Several participants noted that some members of the autistic community imposed their preferences on others. As Participant 279 describes, this imposition may feel worse coming from autistic people (than neurotypical people) as one might expect them to be more accepting of differences, including how someone self‐identifies. Many respondents also reported that *non‐autistic* people (e.g., professionals, parents, teachers, etc.) imposed, particularly person‐first, language on them.

Generally ‘person with autism’ or language like that is used by neurotypical people to speak over autistic people and is generally used in ableist conversations without regard to autistic peoples' opinions. (Participant 381, United States of America, pg. 49, lines 35‐37)

Multiple participants mentioned occasions where their language use was corrected by non‐autistic people (often from ‘autistic person’ to ‘person with autism’). One participant even stated that there had been instances where professionals would correct them if they described themselves as autistic (by calling them a ‘person with autism’). In disregarding their preferences, participants felt that their voices were discounted, their experiences dismissed, and their safety diminished.

### Reflection

As non‐autistic researchers (CTK and HH), our primary aim was to faithfully represent, and amplify, the voices of our autistic participants. In order to so, it was important to recognize the contexts that form the backdrop to our analyses, acknowledge our own positions in the analysis process, and avoid imposing our own (non‐autistic) interpretation on responses (as much as possible). This is particularly pertinent considering contemporary discussions concerning the autistic status of researchers in autism research (Guest, 2020). Through illuminating the architecture of our analysis as well as accounting for our own positions, we hope that the results can add to the growing body of knowledge co‐generated with both autistic and non‐autistic people.

In our qualitative analysis, we took an iterative approach to coding; in discussions with each other, we (HH and CTK) looked to ensure that we were coding all responses and considering all data within theme and subtheme development. To ensure faithful representation of the responses we drew from multiple respondents in the analysis and ensured divergent perspectives were represented. The results presented are informed from a critical realist ontological position and contextualist epistemological position, meaning that we understood the participants as having an ‘authentic reality’ (Rogers & Rogers, 1997), though how this is represented by participants is colored through social and cultural structures of meaning (Braun & Clarke, 2013). We made the decision to adopt this approach to reflexive thematic analysis so that the voices of the participants were prioritized in the write up, and were not obscured by a heavy theoretical analytic strategy.

Whilst we made efforts to ensure the integrity of our analysis it would be remis to not address personal and professional stances relevant to this data. CTK has subject‐specific knowledge and is a co‐founder of the U21 Autism Research Network—a global network aiming to address issues of diversity and inclusion in autism research. HH has methodological specialism and an interest in language preferences and reclamation both within research as well as through personal characteristics. As supporters of identity‐first language and the social model of disability, we found numerous responses that resonated with our perspectives, and many that educated us further by illuminating unforeseen nuances in these debates. We also found responses that diverged; through discussion we explored these responses and ensured they were included in the analytic process and write up. Reflections are not simply accounting for the impact we had, but to also account for the impact the research had on us. Throughout the process we enhanced our understanding of the politics of language preferences; CTK developed a better awareness of language reclamation, and HH developed a better understanding of the neurodiversity movement and how it also synapses into the disability movement.

## DISCUSSION

The current study comprises the first ever cross‐cultural investigation of autism‐related language preferences, and as such, significantly advances our understanding of the preferences of (English‐speaking) autistic adults across the globe. Our findings demonstrate that the most popular terms were similar across all countries studied. The terms “Autism’, ‘Autistic person’, ‘Is autistic’, ‘Neurological/Brain Difference’, ‘Differences’, ‘Challenges’, ‘Difficulties’, ‘Neurotypical people’, and ‘Neurotypicals’ were consistently favored across all countries. Despite relatively high agreement across groups, both our qualitative and quantitative data demonstrate that there is great variability in language preferences within the autistic community, thus supporting Robison's (2019) assertion that ‘language that is appropriate to one person is offensive to another’ (p. 1006). Hence, the overriding principle to follow is to *ask the autistic people one interacts with about their preferences* (Kenny et al., 2016; Mackelprang & Salsgiver, 2009). In scenarios where one is unable to gain clarification on personal preferences (or in scenarios where there are not autistic individuals to defer to), we recommend that individuals consider (a) which terms are endorsed by the majority of the autistic community, and (b) the ideologies underlying certain language choices (e.g., neurodiversity, avoiding ableism, etc.).

In line with this, across all countries, there was a majority preference for identity‐first language. The most popular terms for self‐identifying were ‘autistic person’ (75.9%–88.1% endorsement across countries), ‘neurodivergent person’ (65.2%–75.9%), and ‘autistic’ (65.6%–72.6%). In contrast, there was considerably lower endorsement of person‐first language (e.g., ‘person with autism’; 18.5%–28.6%). Correspondingly, many reasoned that person‐first language should not be used as autism cannot and should not be separated from them, and such language inadvertently conveys that autism is a ‘defect’ to be removed. These sentiments chime with previous work (Bagatell, 2010; Bury et al., 2020; Davidson & Henderson, 2010; Hurlbutt & Chalmers, 2002; Kenny et al., 2016; Botha et al., 2021) and reflect a growing movement amongst activists and scholars arguing against person‐first terminology. Rather, the autistic participants responded that language should be identity‐first as autism is an integral part of who they are, just like their ethnicity, gender or sexuality. This preference for identity‐first language is consistent with prior research conducted in Australia (Bury et al., 2020) and the United Kingdom (Kenny et al., 2016), and with recent recommendations for avoiding ableist language (Bottema‐Beutel et al., 2021).

However, despite relatively high endorsement of ‘autistic’ as a noun (e.g., ‘an autistic’, ‘those autistics’) in our quantitative data, our qualitative results illuminate that this term can be dehumanizing, reducing autistic people to solely their diagnosis. Importantly, these terms, which may have been used previously to marginalize autistic people, have been reclaimed by the community and may not be appropriate for non‐autistic people to use. Reclamation of language has been conceptualized as one way that targeted minorities cope with verbal derogation (e.g., Cervone et al., 2021; Wang et al., 2017), and has been demonstrated in the case of race (e.g., Dea & Saucier, 2020), gender (e.g., Currans, 2017), and sexuality (e.g., Gray, 2009) amongst others. Importantly, Jeshion (2020) identifies two kinds of reclamation—‘pride reclamation’ and ‘insular reclamation’. The former describes reappropriated slurs that express pride and eventually become descriptors used by both the ingroup and outgroup (e.g., ‘queer’, ‘black’). The latter constitutes reappropriated slurs that evoke solidarity within the ingroup in the face of shared oppression, and therefore cannot be used by the outgroup. Since the autistic participants highlight that ‘an autistic’/‘autistics’ should only be used by autistic people, this is an example of insular reclamation. Since we asked participants which terms, *they* would be happy to use (rather than what they would be happy for *others* to use), we may not have detected this insular reclamation in the quantitative data. Future studies could ask the autistic community which terms they would be happy to use themselves, and for others (e.g., neurotypicals or non‐autistic people) to use to talk about autism, thus providing greater insight on what language can be used and by whom. In the meantime, it is paramount that non‐autistic people avoid using ‘autistic’ as a noun, and instead only use it as an adjective (e.g., ‘autistic person’, ‘autistic people’, ‘James is autistic’, etc.).

In the present study, we also found that the term ‘autism’ was consistently the most popular across all country groups (91.1%–96.3% endorsement). In contrast, the terms ‘autism spectrum disorder’ (54.1%–67.7%), ‘autism spectrum condition’ (20.0%–49.1%) and ‘Asperger's syndrome’ (14.7%–31.3% endorsement) were considerably less popular. However, a principal point of contention *between countries* was the use of ‘Asperger's syndrome’ and ‘autism spectrum condition’—both of these terms were more popular in the United Kingdom than in at least one other country. Considering that the linguistic preferences in the United Kingdom (as documented in Kenny et al., 2016) have often been assumed to reflect those globally, it is notable that the preferences in the United Kingdom diverged most from the other five countries studied. At present, it is not entirely clear why there would be higher endorsement of these terms in the United Kingdom. Notably, however, ‘Asperger's syndrome’ was endorsed by the highest proportion of participants in the United Kingdom [31.3%] and Ireland [25.9%], both of which use the International Classification of Diseases (ICD) rather than the Diagnostic and Statistical Manual of Mental Disorders (DSM; which has historically been used in Australia [endorsement:22.2%], Canada [endorsement:14.7%], New Zealand [endorsement:14.8%], and the United States [endorsement:19.3%]). Whilst the diagnosis of Asperger's syndrome was removed from the DSM in 2013 (DSM‐V), it was not until 2019 that Asperger's syndrome was removed from the ICD (ICD‐11). Hence, one potential explanation for the increased popularity of the term ‘Asperger's syndrome’ in the United Kingdom and Ireland is that the diagnostic label persisted longer in these countries. In addition, the higher rates of endorsement for ‘Autism Spectrum Condition’ in the United Kingdom may be caused by the National Health Service (UK) using this term (rather than ‘Autism Spectrum Disorder’) in numerous official documents (e.g., Department of Health, 2015; NHS England, 2019). Despite higher endorsement of these terms in the United Kingdom than in other countries, it is important to note that ‘autism spectrum condition’ (49.1%), and even more so, ‘Asperger's syndrome’ (31.3%) were still relatively unpopular terms.

A general aversion to the term ‘Asperger's’ in the quantitative data was mirrored in the qualitative responses—a large proportion of participants eschewed the use of this term due to its ties with functioning descriptors and eugenics, and it being diagnostically obsolete. However, despite a multitude of participants arguing vehemently against the use of ‘Asperger's’, many highlighted that this term is still used by many autistic individuals for a number of reasons. For example, whilst some individuals may use this this term as they feel it comprises a core part of their identity since their diagnosis, others use it to distance themselves or manage the stigma associated with autism. Most concerningly, however, several participants noted that some use this term to feel ‘superior’ to other autistic people (see De Hooge, 2019 for further discussion of ‘Aspie supremacy’). As highlighted by several participants, this latter manifestation is deeply entrenched in ableism as it suggests that those with higher support needs are ‘inferior’ or less than those with Asperger's. Many participants responded that having this clinical or linguistic distinction between different autistic people damages the unity of the community, and instead generates a hierarchy based on ‘socially‐valued’ differences between autism and Asperger's syndrome.

In line with this, there was wide agreement that functioning labels are divisive, as they unnecessarily segregate autistic people, inaccurate, as so‐called functioning varies across time and situations, and unhelpful, as they lead to ‘high‐functioning’ individuals missing out on support and ‘low‐functioning’ individuals being infantilized or ignored. However, despite the autism community and researchers repeatedly expressing that functioning labels are problematic (e.g., Bottema‐Beutel et al., 2021; Kenny et al., 2016; Pukki et al., 2022; Williams, 2019), a recent article proposed the classification of ‘profound autism’ as an administrative term to apply to autistic individuals with high support needs (Lord et al., 2022). Specifically, this article proposed that this label should be used for those ‘requiring 24 hours access to an adult who can care for them if concerns arise, being unable to be left completely alone in a residence, and not being able to take care of basic daily adaptive needs’ (Lord et al., 2022). The article notes that in most cases, these needs will be associated with intellectual disability, limited language, or both, thus proposing a label to classify the most vulnerable autistic individuals (Lord et al., 2022). This article has been criticized by the *Global Autistic Task Force on Autism Research*—a diverse group of autistic professionals and representatives (including autistic clinicians, therapist, educators, researchers, parents and family members of autistic people, autistic people of color, autistic people from the global south, autistic women and autistic people belonging to gender minorities)‐for multiple reasons (Pukki et al., 2022). Firstly, the authors argue that this term would not be sufficient to steer service provision or research efforts, just as the terms ‘high‐functioning’ and ‘low functioning’ were. As highlighted by Pukki et al. (2022) and our respondents, these terms are not useful as support needs are associated with co‐occurring characteristics and health issues in many combinations, and often fluctuate across time and situations. Secondly, this term gives the false impression that intellectual disability and limited language are core characteristics of autism. An autistic person with these characteristics would labeled as ‘profoundly autistic’, suggesting they are ‘more autistic’ or further down some imaginary linear spectrum, than an autistic person without them. ‘Profoundly autistic’ misleadingly refers to people who have ‘profound impairments’ that are not autism specific, irrespective of their level of autistic characteristics (Pukki et al., 2022). Rather than using ‘high‐functioning’, ‘low‐functioning’ or ‘profound autism’, we suggest that researchers, practitioners, and society more broadly use clear brief descriptions, for example ‘autistic person with intellectual disability’, ‘autistic person with minimal verbal language’, or ‘autistic person with extreme anxiety and a co‐occurring physical condition’ (in line with Bottema‐Beutel et al., 2021; Pukki et al., 2022).

Across each of the country groups, there was also a preference for terms that lead people to consider autism as a part of natural diversity, rather than a deficit. This was demonstrated by a large majority conceptualizing autism as a ‘Neurological/Brain Difference’ (77.0%–87.0%), and high levels of endorsement for the terms ‘differences’ (70.6%–79.5%), ‘difficulties’ (72.2%–80.0%) and ‘challenges’ (67.9%–81.2%). In contrast, there was considerably lower endorsement of medicalised and pathologizing language such as ‘disease’ (0.0%–1.5%), ‘healthy control’ (0.0%–4.4%) and ‘deficits’ (4.7%–14.8%). Correspondingly, in line with the social model of disability, numerous participants highlighted that many of their so‐called ‘difficulties’ arise as a result of being placed within a neurotypical world not built for them, rather than due to being inherently ‘deficient’. Nevertheless, in the current study respondents expressed a range of perspectives on whether autism can be conceptualized as solely a ‘difference’ wherein ‘difficulties’ only arise due to being placed within a neurotypical society. These respondents noted that, for some autistic individuals, difficulties may remain even after suitable accommodations. Thus, describing autism as solely a difference may not only be inaccurate, but also harmful‐ impeding access to services and support (Baker, 2011). These sentiments chime with current conceptualizations of the social model of disability which recognize both internal and external societal factors that may be disabling for individuals (Crow, 1996; Hogan, 2019; Oliver, 2013).

The autistic respondents, however, were not divided in their view that language should be concise, accurate and specific. Whilst numerous participants favored the terms ‘neurodivergent’ and ‘neurotypical’, many also noted that these terms are not synonymous with ‘autistic’ and ‘non‐autistic’ respectively. The term ‘neurodivergent’ encompasses a range of other conditions (e.g., ADHD, dyslexia, etc.) and therefore is not specific to autism. Similarly, the word ‘neurotypical’ rules out other forms of neurodivergence whereas non‐autistic does not; it is possible to be non‐autistic but not neurotypical (e.g., if you have ADHD but are not autistic). In addition, many participants highlighted that the use of these terms was context‐dependent. When referring to their experiences as an autistic person specifically, the participants commented that they would use the term ‘autistic person’. In contrast, when referring to themselves more generally, or when making reference to multiple neurodivergences (e.g., ADHD, dyslexia, anxiety, etc), participants stated that they would use a broader term like ‘neurodivergent person’.

One of the most striking points raised by respondents was that non‐autistic individuals often imposed their language preferences on them. Unfortunately, this tendency for non‐autistic individuals to speak for, and make decisions on behalf of autistic people is also seen more broadly. Autistic people are often excluded from decisions that affect their lives, thus causing them to feel disenfranchised (see Fletcher‐Watson et al., 2019; Nicolaidis et al., 2011; Pellicano et al., 2014a, 2014b; Pellicano & Stears, 2011). It is imperative that researchers, clinicians and practitioners use terminology that autistic people endorse (rather than imposing their own preferences as discussed) in order to fulfill their ethical duty and to maintain their own legitimacy within the autism community (Kenny et al., 2016). Beyond the scientific sphere, the general public also has the responsibility to use language that is accurate, respectful, and chosen by the wider autistic community (or those autistic people close to them).

As stated previously, beyond using the terms endorsed by the autistic community, we recommend that individuals *also* consider the *ideologies* that underlie certain language choices. Language is not simply descriptive but also performative (Botha et al., 2021; Bottema‐Beutel et al., 2021; Sacks, 1992). Through language, we take a particular stance, produce specific versions of oneself and others (Potter & Wetherell, 1987), and perpetuate ideologies (Fairclough, 2013). Hence, the words or phrases we use may influence societal perceptions of autistic people, and shape the identity of individuals themselves (Blaska, 1993; Froschl, 1984; Zola, 1993). Using ableist language can have wide‐ranging negative impacts, for example on disability policy, education, therapeutic practices and social attitudes about autistic people (Billawala & Wolbring, 2014; Bottema‐Beutel et al., 2021; Woods, 2017). On the other hand, using identity‐first language has been associated with greater awareness of, and identification with, the neurodiversity movement (Kapp et al., 2013), a greater sense of autistic identity, and lower internalized autism‐related stigma (Bury et al., in press.). Therefore, it is crucial that we consider underpinning ideologies, and use language that takes a positive stance, thus creating a brighter future for autistic people, together.

### Limitations

Whilst the current study is highly informative with respect to the language preferences of a sample of autistic adults across the globe, further work is needed to understand the preferences of those from more diverse racial, ethnic, cultural, and linguistic backgrounds (Bottema‐Beutel et al., 2021). Notably, the respondents in our survey were predominantly white, highly educated, English‐speaking individuals from developed individualistic countries (e.g., Australia, Canada, Ireland, New Zealand, UK, USA). Hence, our quantitative and qualitative results may not represent the views of autistic people from different backgrounds to those studied. In order to mitigate this limitation, future work should follow the guidelines outlined by Malone et al. (2022). These authors call for the construction of ethnically and racially inclusive research designs, which can be identified through participatory research involving minoritized groups, to promote maximal inclusion of these underrepresented individuals (see Malone et al., 2022). Another potential strategy is to ensure that the research team reflects the diversity of the autistic individuals it aims to represent (e.g., including those from racial, ethnic, and gender minorities; Jones & Mandell, 2020; Malone et al., 2022; Shaia et al., 2020). Such strategies will dismantle barriers to inclusion by helping the research team acknowledge cultural differences and adjust their approach (see Malone et al., 2022 for a full discussion), thus facilitating future investigations into the linguistic preferences of those from more diverse racial, ethnic, and cultural backgrounds.

In addition to these limitations, although we ensured that our questionnaire met the Web Content Accessibility Guidelines (a set of gold‐standard recommendations for web accessibility used across the globe; Caldwell et al., 2008), the extent to which our findings are representative of those with intellectual disabilities and communicative differences is unclear (since we did not ask participants to provide this information). Moreover, our results may not be representative of those without access to a computer/mobile device and internet. As such, we encourage future studies to find new methods to dismantle barriers to inclusion (e.g., posting written versions of surveys to participants, having participants complete the survey over the phone, etc.) in order to investigate the linguistic preferences of a more diverse set of individuals. Despite these limitations with our sample, the overriding principle of asking participants about their preferences is highly applicable across all forms of diversity (i.e., we can (and should) also ask people from different cultures or with communicative differences about their language preferences, etc.).

Fortunately, recent work has addressed one limitation of the current study by shedding light on the language preferences of French‐speaking autistic individuals (see Geelhand et al., in press). This study found a similar pattern of results to the current one, with a high endorsement of the French‐equivalent terms for ‘Autism’, ‘Autistic person’, ‘Is autistic’, ‘Neurological/Brain Difference’, ‘Difficulties’, ‘Differences’, ‘Neurotypicals’, and ‘Neurotypical people’. The largest difference in findings between studies is that ‘Challenges’ (in French, ‘Défis’), ‘Neurodivergent person’ (in French, ‘Personne neurodivergente’) and ‘Is neurodivergent’ were considerably less popular in the French‐speaking (in French, ‘Est neurodivergent’) [Challenges: 34.75%; Neurodivergent person: 46.77%; Is neurodivergent 45.29%] than in the English‐speaking [Challenges: 76.3%; Neurodivergent person: 70.0%; Is neurodivergent: 68.8%]. sample. In addition, there was much higher endorsement of ‘Has a diagnosis of autism’, and considerably lower endorsement of ‘Has autism’ in the French‐speaking [Has a diagnosis of autism: 61.37%; Has autism: 6.28], than the English‐speaking [Has a diagnosis of autism: 37.9%; Has autism: 39.1%] sample. These differences may arise due to linguistic differences in the semantics of these terms (e.g., ‘Défi’ may be used more in the context of ‘taking up a challenge’, such as running a marathon or learning a new skill, rather than experiencing something as a difficulty or ‘challenge’), differences in the frequency of use across regions, or differences in beliefs about, and experiences of, autism. Future work should emulate that of Geelhand et al. (in press) in other languages. By exploring the linguistic preferences of autistic adults across multiple languages (instead of assuming that their preferences are well aligned with English‐speaking preferences), citizens, researchers and governments can ensure that they use the terminology that is most preferred within their region, thus promoting greater harmony and acceptance.

### Conclusion

This study adopted a mixed‐methods approach to explore the linguistic preferences of 654 English‐speaking autistic adults across the globe. We identified that the terms “Autism’, ‘Autistic person’, ‘Is autistic’, ‘Neurological/Brain Difference’, ‘Differences’, ‘Challenges’, ‘Difficulties’, ‘Neurotypical people’, and ‘Neurotypicals’ were the most popular across all countries studied. Despite relative consensus across groups, both our quantitative and qualitative data demonstrate that there is no universally accepted way to talk about autism. Our thematic analysis revealed the reasons underlying participants’ preferences, generating six core themes, and illuminated an important guiding principle‐ to respect personal preferences. Our findings have significant implications for informing practice, research, and language policy worldwide. With respect to the former, researchers and clinicians should defer to the community by asking the autistic people they interact with about their preferences (rather than enforcing their own). With respect to the latter, relevant governments and organizations should update their policy documentation (e.g., the Autism Research Briefing from the UK Parliament, Autism Information from the U.S. Department of Health and Human Services etc.) to be in line with the preferences of the autistic community, thus encouraging appropriate language use amongst researchers, clinicians, and society more broadly.

## Supporting information


**APPENDIX S1.** Supporting information.

## Data Availability

The data that support the findings of this study are available from the corresponding author upon reasonable request.

## Footnotes

^1^
Importantly, this variation in perspectives should not be framed negatively as a ‘a lack of consensus’, but rather a diversity in preferences stemming from fundamental differences in beliefs about, and experiences of, autism.

^2^
Note that these terms conceptualize autism in a novel way rather than just being a direct translation of the word autism (as would be the case for ‘autisme’ which is a direct translation for autism in French).

^3^
Although these terms are often used as synonyms for ‘non‐autistic people’, some of these terms have slight differences in meaning. The term ‘control’ is often used in academic settings to refer to people without the condition under investigation (in this context, non‐autistic individuals) with these individuals serving as a comparison to those with the condition (in this context, autistic individuals). The term ‘allistic’ is a synonym for ‘non‐autistic’. The term ‘neurotypical’ is used to describe individuals with typical neurological development or functioning. Notably, this term is not an exact synonym of ‘non‐autistic’; it is possible to be non‐autistic but not neurotypical (e.g., if you have ADHD; see further discussion in Results and Discussion).

^4^
It is important to note that the word ‘autistic’ was only seen as inappropriate by some participants when used as a noun (e.g., ‘an autistic’, ‘autistics’) rather than an adjective (e.g., ‘Connor is autistic’). Using ‘autistic’ as an adjective (e.g., ‘he/she is autistic’ or ‘they are autistic’) is the most preferred terminology by the community, over ‘has autism’ and ‘has a diagnosis of autism’.
