# Supplementary material for: Autism‐related language preferences of English‐speaking individuals across the globe: A mixed methods investigation
Source: Autism Res. 2022 Dec 6;16(2):406–28. doi: 10.1002/aur.2864 (PMC10946540; doi:10.1002/aur.2864)
Supplement: Supplementary file 1 — APPENDIX S1. Supporting information. [file AUR-16-406-s001.docx]

Supplementary Materials

**Supplementary Materials A**

Table 1. Participants’ responses to the question “What is your ethnic group?”

| **Ethnic Group** | **Number of participants** | **Percentage of participants**  **(%)** |
| --- | --- | --- |
| Aboriginal Australian | 1 | 0.1529052 |
| Arab | 2 | 0.3058104 |
| Ashkenazi-Sephardi | 1 | 0.1529052 |
| Asian Bangladeshi | 1 | 0.1529052 |
| Asian British | 2 | 0.3058104 |
| Asian Filipino | 1 | 0.1529052 |
| Asian Indian | 10 | 1.52905199 |
| Asian Pakistani | 1 | 0.1529052 |
| Australian European | 1 | 0.1529052 |
| Black African | 5 | 0.76452599 |
| Black American | 1 | 0.1529052 |
| Black British | 1 | 0.1529052 |
| Black Caribbean | 1 | 0.1529052 |
| Black, East Asian, White, Indian | 1 | 0.1529052 |
| British Afrikaans | 1 | 0.1529052 |
| Caucasian/Iberian/Black Caribbean | 1 | 0.1529052 |
| Caucasian/White | 7 | 1.07033639 |
| Chinese | 9 | 1.37614679 |
| English, Irish, French, German, Russian, Slovanian | 1 | 0.1529052 |
| German, Scottish, Norwegian, South Canadian Native Chippewa | 1 | 0.1529052 |
| Hispanic/Northern European | 1 | 0.1529052 |
| Irish Jewish | 1 | 0.1529052 |
| Irish, British, German, Jewish | 1 | 0.1529052 |
| Irish/Welsh/Scottish/Indian/Native American | 1 | 0.1529052 |
| Italian/Sardinian/Ashkenazi | 1 | 0.1529052 |
| Jewish | 1 | 0.1529052 |
| Latino and Jewish | 1 | 0.1529052 |
| Latino/Latina/Latinx/Hispanic | 6 | 1.07033639 |
| Metis and White Settler | 1 | 0.1529052 |
| Mexican | 1 | 0.1529052 |
| Middle Eastern and Greek | 1 | 0.1529052 |
| Mixed English, German, and Norwegian background | 1 | 0.1529052 |
| Mixed White and Indigenous/Native American | 1 | 0.1529052 |
| Mixed white, Irish, Scottish, French and Polish | 1 | 0.1529052 |
| Mostly White: French/Irish/German/English ancestry, Shawnee (native North American) | 1 | 0.1529052 |
| Native American | 2 | 0.3058104 |
| New Zealand European | 5 | 0.76452599 |
| New Zealand European and Fijian Indian | 1 | 0.1529052 |
| New Zealand European, New Zealand Māori | 3 | 0.4587156 |
| New Zealand Maori | 1 | 0.1529052 |
| New Zealand Mixed Maori | 1 | 0.1529052 |
| South African | 1 | 0.1529052 |
| South-East Asian | 1 | 0.1529052 |
| Turkish | 1 | 0.1529052 |
| West Indies/Indian | 1 | 0.1529052 |
| White African | 1 | 0.1529052 |
| White Afrikaner | 1 | 0.1529052 |
| White American | 24 | 3.66972477 |
| White American, Greek, Italian | 1 | 0.1529052 |
| White American/Polish | 1 | 0.1529052 |
| White and Asian | 10 | 1.52905199 |
| White and Black African | 3 | 0.4587156 |
| White and Black Caribbean | 5 | 0.76452599 |
| White and Fiji-Indian | 1 | 0.1529052 |
| White and First Nations | 1 | 0.1529052 |
| White and Latine | 1 | 0.1529052 |
| White and Ojibwa | 1 | 0.1529052 |
| White Arab | 2 | 0.3058104 |
| White Ashkenazi | 3 | 0.4587156 |
| White Ashkenazi Jewish | 2 | 0.3058104 |
| White Asia Minor Greek/British | 1 | 0.1529052 |
| White Australian | 18 | 2.75229358 |
| White Australian/British | 1 | 0.1529052 |
| White Belgian | 2 | 0.3058104 |
| White British and Aboriginal Australian | 1 | 0.1529052 |
| White British/French, Metis | 1 | 0.1529052 |
| White British/Northern European | 1 | 0.1529052 |
| White Canada/Metis | 1 | 0.1529052 |
| White Canadian | 9 | 1.37614679 |
| White Czech | 1 | 0.1529052 |
| White Danish | 2 | 0.3058104 |
| White Dutch | 4 | 0.6116208 |
| White Eastern European | 3 | 0.4587156 |
| White Eastern European/Jewish | 1 | 0.1529052 |
| White English and Ashkenazi Jewish | 1 | 0.1529052 |
| White English and Ukranian | 1 | 0.1529052 |
| White English/Mediterranean | 1 | 0.1529052 |
| White English/Welsh/Scottish/German/Eastern European mix | 1 | 0.1529052 |
| White English/Welsh/Scottish/Northern Irish/British | 252 | 38.5321101 |
| White English/Welsh/Scottish/Northern Irish/British, Western European, and Italian | 1 | 0.1529052 |
| White Estonian | 1 | 0.1529052 |
| White European | 7 | 1.07033639 |
| White European American | 2 | 0.3058104 |
| White European and Scandinavian | 1 | 0.1529052 |
| White Finnic | 1 | 0.1529052 |
| White Finnish/ French Canadian | 1 | 0.1529052 |
| White French | 3 | 0.4587156 |
| White French-Canadian | 1 | 0.1529052 |
| White French/English and White Portuguese/Spanish | 1 | 0.1529052 |
| White French/German/Spanish | 1 | 0.1529052 |
| White German | 4 | 0.6116208 |
| White German American | 1 | 0.1529052 |
| White German and Polish | 1 | 0.1529052 |
| White German/Canadian | 1 | 0.1529052 |
| White Greek | 4 | 0.6116208 |
| White Greek Australian | 1 | 0.1529052 |
| White Honduran | 1 | 0.1529052 |
| White Icelandic | 1 | 0.1529052 |
| White Irish | 88 | 13.4556575 |
| White Irish American | 1 | 0.1529052 |
| White Irish German | 1 | 0.1529052 |
| White Irish Traveller | 1 | 0.1529052 |
| White Irish-Italian & Native American | 1 | 0.1529052 |
| White Italian | 1 | 0.1529052 |
| White Italian/Dutch/Canadian | 1 | 0.1529052 |
| White Jewish | 2 | 0.3058104 |
| White Latine | 1 | 0.1529052 |
| White Mediterranean | 2 | 0.3058104 |
| White Mexican | 1 | 0.1529052 |
| White Mixed European | 1 | 0.1529052 |
| White Mixed Northern/Western European descent | 1 | 0.1529052 |
| White Mixed origin | 1 | 0.1529052 |
| White Mixed Western/Northern European | 1 | 0.1529052 |
| White Neurodivergent | 1 | 0.1529052 |
| White New Zealand European/ Pakeha | 20 | 3.05810398 |
| White Nordic | 1 | 0.1529052 |
| White Northern European | 1 | 0.1529052 |
| White Polish and English, Scottish | 1 | 0.1529052 |
| White Polish/English/Irish & Ashkenazi | 1 | 0.1529052 |
| White Polish/Slavic | 5 | 0.76452599 |
| White Roma | 2 | 0.3058104 |
| White Russian | 1 | 0.1529052 |
| White Scandinavian | 4 | 0.6116208 |
| White South African | 9 | 1.37614679 |
| White Spanish | 1 | 0.1529052 |
| White Swiss and South African | 1 | 0.1529052 |
| White Ukranian | 1 | 0.1529052 |
| White Ukranian German | 1 | 0.1529052 |
| White Undefned/Unknown | 7 | 1.07033639 |
| White, Black Caribbean, and Caribbean Indigenous | 1 | 0.1529052 |
| White, Black Caribbean; Hispanic | 1 | 0.1529052 |
| White, Middle Eastern | 1 | 0.1529052 |
| White, mixed ancestry | 2 | 0.3058104 |
| Wiradjuri | 1 | 0.1529052 |
| Undisclosed | 8 | 1.22324159 |
| **Total** | **654** | **100** |

**Supplementary Materials B-** Language Preferences Questionnaire (Keating et al., 2022)

We have selected most of the autism-related terminology in this questionnaire from a previous study (Kenny et al., 2015). We have also added some additional terms that have previously been used in the scientific literature to talk about autism. Please note that the inclusion of any terms is not a reflection of the opinions of our research team. We are just interested in getting your insight so we know how it is best to talk about autism in science and more broadly in society.

Q1a. Please select which terms you are **happy to use** to talk about autism. You can select as many as you like.

- Asperger's syndrome
- Autism
- Autism Spectrum Condition (ASC)
- Autism Spectrum Disorder (ASD)
- Other ________________________________________________

Q1b. Please now select your **favourite** term to talk about autism.

- Asperger's syndrome
- Autism
- Autism Spectrum Condition (ASC)
- Autism Spectrum Disorder (ASD)
- Other ________________________________________________

Q2a. Please select which terms you are **happy to use** to describe yourself/ refer to someone else with autism. You can select as many as you like.

- Aspie (e.g., 5 aspies participated in this study)
- Autistic (e.g., 5 autistics participated in this study)
- Autistic person (e.g., 5 autistic people participated in this study)
- Neurodivergent person (e.g., 5 neurodivergent people participated in this study)
- Person on the autism spectrum (e.g., 5 people on the autism spectrum participated in this study)
- Person with autism/ autism spectrum disorder/ autism spectrum condition (e.g., 5 people with autism participated in this study)
- Other ________________________________________________

Q2b. Please now select your **favourite** term to describe yourself/ refer to someone else with autism.

- Aspie (e.g., 5 aspies participated in this study)
- Autistic (e.g., 5 autistics participated in this study)
- Autistic person (e.g., 5 autistic people participated in this study)
- Neurodivergent person (e.g., 5 neurodivergent people participated in this study)
- Person on the autism spectrum (e.g., 5 people on the autism spectrum participated in this study)
- Person with autism/ autism spectrum disorder/ autism spectrum condition (e.g., 5 people with autism participated in this study)
- Other ________________________________________________

Q3a. Please select which terms you are **happy to use** to say that someone has an autism diagnosis. You can select as many as you like.

- Has a diagnosis of autism/ autism spectrum disorder/ autism spectrum condition/Asperger's (e.g., He/She has a diagnosis of autism, They have a diagnosis of autism )
- Has autism/ Has Asperger's (e.g., He/She has autism, They have autism)
- Is autistic/Is Aspergic (e.g., He/She is autistic, They are autistic)
- Is neurodivergent (e.g., He/She is neurodivergent, They are neurodivergent)
- Other ________________________________________________

Q3b. Please select your **favourite** term to say that someone has an autism diagnosis.

- Has a diagnosis of autism/ autism spectrum disorder/ autism spectrum condition/Asperger's (e.g., He/She has a diagnosis of autism, They have a diagnosis of autism)
- Has autism/Has Asperger's (e.g., He/She has autism, They have autism)
- Is autistic/Is Aspergic (e.g., He/She is autistic, They are autistic)
- Is neurodivergent (e.g., He/She is neurodivergent, They are neurodivergent)
- Other ________________________________________________

Q4a. Please select which terms you are **happy to use** when talking about autism more broadly. You can select as many as you like.

- Condition
- Disability
- Disease
- Disorder
- Neurological/Brain Difference
- Other ________________________________________________

Q4b. Please select your **favourite** term to talk about autism more broadly.

- Condition
- Disability
- Disease
- Disorder
- Neurological/Brain Difference
- Other ________________________________________________

Q5a. Please select which terms you are **happy to use** to talk about the challenges associated with autism. You can select as many as you like.

- Challenges
- Deficits
- Differences
- Difficulties
- Impairments
- Lower/Higher performance
- Poorer/Better performance
- Other ________________________________________________

Q5b. Please select your **favourite** term to talk about the challenges associated with autism.

- Challenges
- Deficits
- Differences
- Difficulties
- Impairments
- Lower/Higher performance
- Poorer/Better performance
- Other ________________________________________________

Q6a. Please select which terms you are **happy to use** when talking about people**without** a diagnosis of autism. You can select as many as you like.

- Allistic people
- Allistics
- Control participants
- Controls
- Healthy Controls
- Neurotypical people
- Neurotypicals
- Non-autistic people
- Non-autistics
- Typical people
- Typically developing people
- Other ________________________________________________

Q6b. Please select your **favourite** term to talk about people without a diagnosis of autism.

- Allistic people
- Allistics
- Control participants
- Controls
- Healthy Controls
- Neurotypical people
- Neurotypicals
- Non-autistic people
- Non-autistics
- Typical people
- Typically developing people
- Other ________________________________________________

Q7a. You now have the chance to tell us a bit more about your autism-related language preferences. You could perhaps give reasons for why you like or dislike certain terms. Please type your response in the box below. Note that you can click and drag the right-hand corner down to make the text box larger.

________________________________________________________________

________________________________________________________________

________________________________________________________________

________________________________________________________________

________________________________________________________________

**Supplementary Materials C-** Confirming the veracity of survey responses.

In order to confirm the veracity of the survey responses, we completed a number of quality checks. Firstly, we ensured that there were no responses from the same IP address or GPS coordinates (i.e., longitude and latitude). Following this, we discarded any responses that were incomplete, or were completed in less than 5 minutes (7.5 second per question). We based this threshold on VERSTA’s guidelines for conducting online surveys which suggests that it takes 7.5 seconds on average to answer a question (see https://verstaresearch.com/newsletters/how-to-estimate-the-length-of-a-survey/). In addition, we tested whether this threshold was appropriate by having numerous experimenters complete the survey quickly but truthful to their own preferences. In addition, we checked for any implausible responses (e.g., answering that they got their diagnosis when they were 27 when they have said their age is 21) and excluded these. Finally, we also implemented bot detection in our Qualtrics survey in the form of a reCAPTCHA check. As such, participants were only able to participate if they could prove they were not a robot. We are confident that these measures have enabled us to get valid responses. In addition, the large number of high-quality, and nuanced, responses to the qualitative component of the survey gives us further confidence in the veracity of our data.

**Supplementary Materials D-** Analysis of ‘favorite’ autism-related terminology

*Preferences in the global sample*

In order to explore the percentage of participants who selected each of the autism-related terms as their favorite, we conducted 6 one-way ANOVAs. In the first ANOVA, we assessed language preferences relating to the nomenclature of autism. This identified a significant main effect [*F*(1.85, 1208.52) = 508.42 *p* < .001, *η_P_^2^* = .44]: the term that was selected as favorite by the highest percentage of participants was ‘Autism’ [70.9%], followed by ‘Autism Spectrum Disorder’ [14.1%], followed by ‘Asperger’ [5.8%] and ‘Autism Spectrum Condition’ [2.9%]. The second ANOVA assessed preferences relating to the self/person. This revealed a significant main effect [*F*(2.80, 1828.61) = 124.69, *p* < .001, *η_P_^2^* = .16]: the term that was favored by the highest proportion of the participants was ‘Autistic person’ [40.4%] and ‘Autistic’ [31.7%], followed by ‘Neurodivergent person’ [9.5%], followed by ‘Person on the spectrum’ [5.4%], ‘Aspie’ [4.3%], and ‘Person with Autism/Autism Spectrum Disorder/Autism Spectrum Condition’ [4.1%]. In the third ANOVA, we assessed language preferences for referring to someone with autism. This found a significant main effect [*F*(2.20, 1449.49) = 333.48, *p* < .001, *η_P_^2^* = .34]: ‘Is autistic’ was favored by the highest percentage of participants [65.4%], followed by ‘Has Autism/ Autism Spectrum Disorder/ Autism Spectrum Condition’ [11.5%] and ‘Is neurodivergent’ [10.4%], and finally ‘Has a diagnosis of Autism/ Autism Spectrum Disorder/ Autism Spectrum Condition’ [6.3%]. The fourth ANOVA, which assessed language preferences relating to how autism is conceptualized more broadly, identified a main effect [*F*(2.53, 1649.51)= 134.71, *p* < .001, *η_P_^2^* = .17]. The term that was selected as favorite by the highest percentage of participants was ‘Neurological/Brain Difference’ [46.5%], followed by ‘Disability’ [20.8%], followed by ‘Condition’ [14.1%], followed by ‘Disorder’ [8.4%], and finally ‘Disease’ [0.5%]. In the fifth ANOVA, we assessed language preferences relating to how we talk about the difficulties of autistic people. This identified a significant main effect [F(2.57, 1680.05) = 140.05, p < .001, *η_P_^2^* = .18], revealing that ‘Differences’ [37.6%] was favored by the highest percentage of participants followed by ‘Difficulties’ [24.6%] and ‘Challenges’ [24.3%], followed by ‘Impairments’ [2.6%], ‘Deficits’ [1.5%], ‘Lower/Higher Performance’ [1.1 %], and ‘Poorer/Better Performance’ [0.8%] Finally, in the sixth ANOVA, which assessed preferences relating to how we refer to non-autistic people, we found a significant main effect [*F*(4.43, 2891.72) = 87.07, *p* < .001, *η_P_^2^* = .12]. The terms that were selected as favorite by the highest percentage of participants were ‘Neurotypicals’ [28.9%] and ‘Neurotypical people’ [23.7%], followed by ‘Non-autistic people’ [16.7%] and ‘Allistic people’ [10.1%], followed by ‘Allistics’ [9.0%], followed by ‘Non-autistics’ [3.7%] and ‘Typical people’ [2.3%], ‘Controls’ [1.1%], ‘Control participants’ [0.5%], ‘Typically developing people’ [0.5%] and ‘Healthy Controls’ [0.2%].

*Comparing favorite autism-related language across countries*

As per our pre-registration, for our analyses comparing language preferences across countries, we only included the data from country groups that had at least 50 participants (in which we had sufficient data to draw comparisons between groups). We conducted 6 mixed ANOVAs to examine differences in the favorite terms for each question selected across countries. This included the between-subjects factor *country* (Australia, Canada, Ireland, New Zealand, United Kingdom, United States of America), and the within-subjects factor *term*. In the first ANOVA, which assessed language preferences relating to the nomenclature of autism, we identified a significant main effect of *term* [*F*(1.86, 1077.69) = 402.82, *p* <. 001, *η_P_^2^* = .41] but no significant term x country interaction [*p* = .282]. The lack of this interaction suggests that there were similar patterns of favored terms selected across country groups. The second ANOVA compared preferences relating to the self/person across countries. This revealed that there was a significant main effect of term [*F*(2.75, 1591.81) = 110.81, *p* < .001, *η_P_^2^* = .16], and a term x country interaction [*F*(13.75, 1591,81) = 1,86 *p* = .027, *η_P_^2^* = .02]. Bonferroni-corrected pairwise comparisons demonstrated that a significantly higher percentage of participants in Canada selected ‘Neurodivergent person’ as their favorite term [13.8%] than in the UK [0.9%; *t*(219) = 3.79, *p*bonf = .015, mean difference = 12.9%]. There were no other significant differences (after Bonferroni-correction) between countries for other terms. In the third ANOVA, we compared language preferences for referring to someone with autism. This found a significant main effect of term [*F*(2.22, 1282.41) = 279.08, *p* < .001, *η_P_^2^* = .33], but no term x country interaction [*p* = .096], suggesting similar favored term selections across countries. The fourth ANOVA, which compared the language preferences relating to how autism is conceptualized more broadly across countries, identified a main effect of term [*F*(2.50, 1449.35) = 114.31, *p* < .001, *η_P_^2^* = .17] but no term x country interaction [*p* = .139], thus demonstrating a similar pattern of responses across country groups. In the fifth ANOVA, we compared language preferences relating to how we talk about the difficulties of autistic people across country groups. This identified a significant main effect of term [*F*(2.58, 1492.18) = 114.82, *p* < .001, *η_P_^2^* = .17], but no term x country interaction [*p* = .631]. In the sixth and final ANOVA, which compared preferences relating to how we refer to non-autistic people across countries, we found a significant main effect [*F*(4.43, 2563.06) = 71.88, *p* < .001, *η_P_^2^* = .11], but no term x country interaction [*p* = .143]. Hence, there were similar patterns of responses with respect to favorite autism-related terminology across the country groups.

**Supplementary Materials E-** Analyses comparing language preferences across genders.

In our analysis comparing language preferences across genders, we only included the data from gender groups that had at least 50 participants (in which we had sufficient data to draw comparisons between groups). Therefore, we compared the language preferences of those that identified as cisgender females (n = 248), as non-binary/third gender (n = 163) and cisgender males (n = 113).

In our first ANOVA, which compared preferences relating to the nomenclature of autism, we identified a significant term x gender interaction [F(5.51, 1435.09) = 11.39, p < .001, ηP2 = .042]. Unpacking this interaction using a simple effects analysis [F(2,521) = 33.98, p < .001, ηP2 = .115] revealed that a higher proportion of cisgender males endorsed the term “Asperger’s syndrome” [51.3%] than cisgender females [24.6%], and those that identified as non-binary/third gender [9.8%]. There were no other significant differences (all p > .05).

In our second ANOVA comparing preferences for referring to the self/person, we found a significant term x gender interaction [F(8.41, 2190.01) = 11.31, p < .001, ηP2 = .042]. To unpack this interaction, we conducted simple effects analyses which indicated that percentage endorsement differed across genders for “Aspie” [F(2, 521) = 11.13, p < .001, ηP2 = .041], “Autistic person” [F(2, 521) = 7.16, p < .001, ηP2 = .027], “Neurodivergent person” [F(2, 521) = 21.94, p < .001, ηP2 = .078], “Person on the spectrum” [F(2, 521) = 3.58, p = .029, ηP2 = .014] and “Person with Autism/Autism Spectrum Disorder/Autism Spectrum Condition” [F(2, 521) = 7.32, p < .001, ηP2 = .027]. The term “Aspie” was more popular with cisgender males [33.6%] than cisgender females [19.4%] and those that identify as non-binary/third gender [11.0%]. In contrast, “Autistic person” was more popular with those that identified as non-binary/third gender [85.3%] and cisgender females [78.6%] than cisgender males [66.4%]. The term “Neurodivergent person” was most popular with those that identify as non-binary/third gender [85.3%], followed by cisgender females [66.1%], followed by cisgender males [49.6%]. “Person on the spectrum” was most popular with cisgender males [41.6%] followed by cisgender females [31.9%], followed by those that identify as non-binary/third gender [26.4%]. Finally, the term “Person with autism/autism spectrum disorder/autism spectrum condition” was more popular with cisgender males [36.3%] than with cisgender females [23.4%] and non-binary/third gender individuals [16.6%].

In our third ANOVA, which compared preferences for referring to autistic identity, we found a significant term x gender interaction [F(5.64, 1469.43) = 11.36, p < .001, ηP2 = .042]. To unpack this interaction, we conducted simple effects analyses which indicated that percentage endorsement varied across genders for “Has a diagnosis of Autism/Autism Spectrum Disorder/Autism Spectrum Condition” [F(2,521) = 6.23, p = .002, ηP2 = .023] and “Is neurodivergent” [F(2,521) = 20.36, p < .001, ηP2 = .072]. The term “Has a diagnosis of Autism/Autism Spectrum Disorder/Autism Spectrum Condition” was more popular with cisgender females [43.5%] and cisgender males [41.6%] than those that identified as non-binary/third gender [27.0%]. In contrast, the term “Is neurodivergent” was most popular with non-binary/third gender people [80.4%] followed by cisgender females [68.5%] followed by cisgender males [45.1%].

In our fourth ANOVA comparing preferences for referring to autism more broadly, we found a significant term x gender interaction [F(7.02, 1829.22) =5.91, p < .001, ηP2 = .022]. To unpack this interaction, we conducted simple effects analyses which indicated that percentage endorsement differed across genders for “Disability” [F(2,521) = 9.52, p < .001, ηP2 = .035] and “Neurological/Brain Difference” [F(2,521) = 6.46, p = .002, ηP2 = .024]. The term “Disability” was more popular with those that identified as non-binary/third gender [68.7%] and cisgender females [61.7%] than cisgender males [43.4%]. Similarly, “Neurological/Brain Difference” was more popular among those that identified as non-binary/third gender [84.7%] and cisgender females [80.2%] than cisgender males [67.3%].

In our fifth ANOVA, which compared preferences for referring to the difficulties of autistic people, we found a significant term x gender interaction [F(10.23, 2664.18) = 4.87, p < .001, ηP2 = .018]. To unpack this interaction, we conducted simple effects analyses which indicated that percentage endorsement varied across genders for “Challenges” [F(2,521) = 4.58, p = .011, ηP2 = .017], “Deficits” [F(2,521) = 3.70, p = .025, ηP2 = .014], “Difficulties” [F(2,521) = 5.95, p = .003 ηP2 = .022], “Lower/Higher performance” [F(2,521) = 4.75, p = .009, ηP2 = .018], and “Poorer/Better performance” [F(2,521) = 5.39 p = .005, ηP2 = .020]. The term “Challenges” was more popular amongst those that identify as non-binary/third gender [80.4%] and cisgender females [75.8%] than cisgender males [64.6%]. Conversely, the term “Deficits” was more popular among cisgender males [19.5%] than cisgender females [10.9%] and non-binary/third gender people [9.2%]. “Difficulties” was more popular among non-binary/third gender people [80.4%] and cisgender females [76.6%] than cisgender males [62.8%]. “Lower/Higher performance” was more popular among cisgender males [23.0%] than cisgender females [12.9%] and non-binary/third gender individuals [10.4%]. Similarly, “Poorer/Better performance” was more popular among cisgender males [20.4%%] than cisgender females [10.5%] and non-binary/third gender individuals [8.0%].

In our sixth and final ANOVA, which compared preferences for referring to non-autistic people, we also found a significant term x gender interaction [F(12.78, 3329.34) = 10.10, p < .001, ηP2 = .037]. To unpack this interaction, we conducted simple effects analyses which indicated that percentage endorsement varied across genders for “Allistic people” [F(2,521) = 36.04, p < .001, ηP2 = .122], “Allistics” [F(2,521) = 31.80, p < .001, ηP2 = .109], “Healthy controls” [F(2,521) = 3.89 p = .021, ηP2 = .015], “Neurotypicals” [F(2,521) = 5.80, p = .003, ηP2 = .022], “Non-autistic people” [F(2,521) = 3.40, p = .034, ηP2 = .013], and “Typical people” [F(2,521) = 5.20, p = .006, ηP2 = .020]. The terms “Allistic people” and “Allistics” were most popular with non-binary/third gender people [Allistic people: 70.6%; Allistics: 64.4%], followed by cisgender females [Allistic people: 39.9%; Allistics: 36.3%], followed by cisgender males [Allistic people: 24.8%; Allistics: 21.2%]. The term “Healthy controls” and “Typical people” were more popular with cisgender males [Healthy Controls: 5.3%; Typical people: 6.2%] than with cisgender females [Healthy Controls: 1.6%; Typical people: 1.6%] and non-binary/third gender individuals [Healthy Controls: 0.6%; Typical people: 0.6%]. Finally, the terms “Neurotypicals” and “Non-autistic people” was more popular with non-binary/third gender individuals [Neurotypicals: 81.0%; Non-autistic people: 69.3%] and cisgender females [Neurotypicals: 74.2%; Non-autistic people: 63.3%] than with cisgender males [Neurotypicals: 62.8%; Non-autistic people: 54.0%].

**Supplementary Materials F-** Analyses comparing the language preferences of formally diagnosed and self-identified autistic individuals.

In the following analyses, we compare the preferences of formally diagnosed and self-identified autistic individuals. In our ANOVAs assessing preferences relating to (a) the nomenclature of autism, (b) conceptualising autism more broadly, (c) the difficulties of autistic people, and (d) referring to non-autistic people, there was not a significant interaction between term and diagnostic group [all p > .05]. This suggests that percentage endorsement was similar between formally diagnosed and self-identified autistic individuals for terms within these categories.

However, there was a weak significant interaction between term and diagnostic group in our ANOVAs assessing preferences for e) referring to the self/person [F(4.10, 2671.90) = 2.90, p = .020, η_P_^2^ = .004], and f) for referring to someone’s autistic identity [F(2.74, 1784.69) = 3.73, p = .011, η_P_^2^ = .006]. Unpacking these interactions revealed that there was a higher endorsement of the term ‘Autistic’ (e.g., ‘an autistic’, ‘those autistics’) in formally diagnosed [69.6%] than self-identified autistic individuals [60.8%; [t(652) =-2.06, p_bonf_ = .040, mean difference = -8.8%]. In contrast, there was significantly higher endorsement of the term “Is neurodivergent” in the self-identified [mean(SEM) = 77.2%] than the formally diagnosed [67.7%] participants after Bonferroni-correction [t(=652) = 2.27, p_bonf_ = .024, mean difference = 9.5%]. Finally, perhaps unsurprisingly there was higher endorsement of the term ‘Has a diagnosis of Autism/Autism Spectrum Disorder/Autism Spectrum Condition’ in the formally diagnosed [40.3%] than the self-identified [30.4%] participants after Bonferroni-correction [t(652) = -2.25, p = .025, mean. Difference = 9.9%]. There were no other significant differences in percentage endorsement between these participant groups.

**Supplementary Materials G-** Number of respondents from each country included in our thematic analysis.

Table 2. Number of participants from each country included in our thematic analysis

| **Country** | **Number of participants** | **Percentage of participants (2dp)** |
| --- | --- | --- |
| Australia | 57 | 13.77% |
| Austria | 1 | 0.24% |
| Belgium | 1 | 0.24% |
| Canada | 72 | 17.39% |
| Denmark | 1 | 0.24% |
| Finland | 1 | 0.24% |
| France | 1 | 0.24% |
| Germany | 7 | 1.69% |
| Greece | 1 | 0.24% |
| Honduras | 1 | 0.24% |
| Hong Kong (S.A.R.) | 1 | 0.24% |
| Iceland | 1 | 0.24% |
| India | 1 | 0.24% |
| Ireland | 55 | 13.29% |
| Jamaica | 2 | 0.48% |
| Kenya | 1 | 0.24% |
| Mexico | 1 | 0.24% |
| the Netherlands | 6 | 1.45% |
| New Zealand | 32 | 7.73% |
| Norway | 1 | 0.24% |
| Romania | 1 | 0.24% |
| South Africa | 14 | 3.38% |
| Sweden | 1 | 0.24% |
| Switzerland | 1 | 0.24% |
| UK | 65 | 15.70% |
| USA | 87 | 21.01% |
| Vanuatu | 1 | 0.24% |
